# Linking gas and particle ejection dynamics to boundary conditions in scaled shock-tube experiments

**DOI:** 10.1007/s00445-021-01473-0

**Published:** 2021-07-20

**Authors:** Valeria Cigala, Ulrich Kueppers, Juan José Peña Fernández, Donald B. Dingwell

**Affiliations:** Ludwig-Maximilians-Universität (LMU) Munich, Theresienstr 41, 80333 Munich, Germany

**Keywords:** Explosive volcanism,, Experimental volcanology,, Spreading angle,, Shock-tube

## Abstract

Predicting the onset, style and duration of explosive volcanic eruptions remains a great challenge. While the fundamental underlying processes are thought to be known, a clear correlation between eruptive features observable above Earth’s surface and conditions and properties in the immediate subsurface is far from complete. Furthermore, the highly dynamic nature and inaccessibility of explosive events means that progress in the field investigation of such events remains slow. Scaled experimental investigations represent an opportunity to study individual volcanic processes separately and, despite their highly dynamic nature, to quantify them systematically. Here, impulsively generated vertical gas-particle jets were generated using rapid decompression shock-tube experiments. The angular deviation from the vertical, defined as the “spreading angle”, has been quantified for gas and particles on both sides of the jets at different time steps using high-speed video analysis. The experimental variables investigated are 1) vent geometry, 2) tube length, 3) particle load, 4) particle size, and 5) temperature. Immediately prior to the first above-vent observations, gas expansion accommodates the initial gas overpressure. All experimental jets inevitably start with a particle-free gas phase (gas-only), which is typically clearly visible due to expansion-induced cooling and condensation. We record that the gas spreading angle is directly influenced by 1) vent geometry and 2) the duration of the initial gas-only phase. After some delay, whose length depends on the experimental conditions, the jet incorporates particles becoming a gas-particle jet. Below we quantify how our experimental conditions affect the temporal evolution of these two phases (gas-only and gas-particle) of each jet. As expected, the gas spreading angle is always at least as large as the particle spreading angle. The latter is positively correlated with particle load and negatively correlated with particle size. Such empirical experimentally derived relationships between the observable features of the gas-particle jets and known initial conditions can serve as input for the parameterisation of equivalent observations at active volcanoes, alleviating the circumstances where an a priori knowledge of magma textures and ascent rate, temperature and gas overpressure and/or the geometry of the shallow plumbing system is typically chronically lacking. The generation of experimental parameterisations raises the possibility that detailed field investigations on gas-particle jets at frequently erupting volcanoes might be used for elucidating subsurface parameters and their temporal variability, with all the implications that may have for better defining hazard assessment.

**Supplementary Information:**

The online version contains supplementary material available at 10.1007/s00445-021-01473-0.

## Introduction

During short-lived, transient stages of Strombolian, Vulcanian and (in part) Plinian events at explosive volcanic centres, two-phase mixtures (solid pyroclasts and lithics, plus gas) are impulsively ejected in the atmosphere at initial velocities that can range from tens to hundreds of m/s. These jets can be initially subsonic (Mach number *M* < 1) or supersonic (*M* > 1) (Kieffer and Sturtevant 1984; Koyaguchi et al. 2010; Carcano et al. 2014).

Analyses of direct observations of explosive eruptions have employed particle ejection properties, like velocity and trajectory, to estimate prior-to-ejection parameters, such as vent geometry, ‘the depth of origin’ of the ejected pyroclasts, as well as post-ejection parameters such as maximum range (Bombrun et al. 2014; Dürig et al. 2015; Gaudin et al. 2016; Bernard 2018; Salvatore et al. 2018). The findings of such studies have been integrated into models of past eruptions (Konstantinou 2015) to validate source conditions (Bombrun et al. 2015; Tournigand et al. 2017; Salvatore et al. 2018) and to assist in the generation of probabilistic hazard maps (De Michieli Vitturi et al. 2010; Alatorre-Ibargüengoitia et al. 2012). The particle and gas components ejected exhibit variable degrees of coupling which in turn yield variable ejection dynamics. The trajectories of large (cm to m size) particles can be analysed as a ballistic problem (Mastin 2001; Bertin 2017). Finer particles are typically studied in the context of the gas thrust versus buoyant regimes of plume dynamics (Patrick 2007; Tournigand et al. 2017). At active volcanoes, the characteristics of gas and particle ejection may be amenable to continuous monitoring whereby real-time information on source conditions may be inferred. In detail, each eruption complicates predictions of the next because it potentially alters the vent geometry, either destructively (vent size increases due to explosion intensity, erosion and/or slumping) or constructively (re-shaping the vent by deposition of loose or partially welded pyroclastic material). Thus, there is a growing number of studies that investigate the geometry and morphology of volcanic vents (e.g. Turner et al. 2017; Salvatore et al. 2018; Schmid et al. 2020) and their evolution during activity from the experimental point of view (Solovitz et al. 2014; McNeal et al. 2018).

Field observations of the complex dynamics of volcanic jets and plumes can be complemented by theoretical analysis, numerical modelling and experimental investigation. Morton et al. (1956) provided an early theoretical basis for the analysis of volcanic jets and plumes. The particle-gas mixture has been commonly treated as a pseudogas (Kieffer and Sturtevant 1984; Turcotte et al. 1990; Woods 1995)—an approximation which implies perfect phase coupling, such that the bulk properties can be described as a function of the gas fraction together with the particle fraction in thermal equilibrium with the gas phase. Thermal equilibrium is commonly assumed for particles < 0.5 mm (Woods 1995). Advances in the understanding of phase relationships and in computational power, have enabled gas-particle dynamics to be approached as more complex two-phase (Bercovici and Michaut 2010) and multiphase systems (Neri et al. 2003) and more complex coupling regimes (Carcano et al. 2013; Cerminara et al. 2016).

Shock-tube experiments are commonly used to provide further insights into the gas and particle fragmentation and ejection dynamics (e.g. Kieffer and Sturtevant 1984; Chojnicki et al. 2006; Alatorre-Ibargüengoitia et al. 2010) and column entrainment dynamics (e.g. Carey et al. 1988; Jessop and Jellinek 2014; Jessop et al. 2016) within volcanic context. We are interested in particular in the near-vent ejection dynamics. Commonly, experimental investigation of gas-particle jets in the engineering literature focus on non-transient (i.e. steady-state) regimes. Sommerfeld (1994), for example, studied the structure of underexpanded (i.e. where the jet maintains a pressure higher than the surrounding atmosphere), quasi-steady, supersonic gas-particle jets. In that study, the solid phase (quantified in terms of a variable particle number density upon exit) was composed of glass beads with particle diameters (*d*_*p*_) of 26 and 45 μm. The experimental facility incorporated a gas-particle mixing chamber and a feeder to adjust and control the particle mass flow rate. The particle spreading angle was defined and measured as the angular deviation from a vertical trajectory, similar to the definition of ‘spread angle’ by Head and Wilson (1989). Observations of spreading angle in previous studies show (1) a weak positive correlation of spreading angle with particle number density at exit; (2) a slight negative correlation of jet spreading with reservoir-to-ambient pressure ratio; and (3) a negative correlation of spreading angle with particle size, interpreted as being due to a stronger coupling with the gas phase (Sommerfeld 1994). Orescanin and Austin (2010) experimentally investigated the characteristics of underexpanded gas jets from finite reservoirs (non-dimensional volume from 1 to 26) and characterized their impulsive and transient dynamic behaviour. They found that for reservoir-to-ambient pressure ratios greater than 15, jet characteristics, such as Mach disk location, can be predicted by the theoretical equations describing jets from infinite reservoirs. They later applied their findings to investigate the supersonic nature of short-lived explosive eruptions (Orescanin et al. 2010). Chojnicki et al. (2006) performed shock-tube experiments and investigated the behaviour of rapidly decompressed gas and particle mixtures and associated shock waves. They used monodisperse mixtures of glass spheres (*d*_*p*_ = 45–150 μm, and density *ρ*_*p*_ = 2500 kg/m^3^) and air that were rapidly decompressed at pressure ratios between the reservoir and ambient (*P*_*r*_/*P*_*a*_) varying from 1 to 70. They found that the presence of particles significantly reduced shock velocities (by 30–40%) and strengths (by 60%) compared to the predictions made with the pseudogas approximation. Moreover, the approximation overpredicted the mixture velocity and they suggested that the difference was due to imperfect coupling between the phases. Salvatore et al. (2020) used a 3-m-long transparent shock-tube apparatus with vent geometries like the one used in our study, but other experimental conditions differed (for example, the maximum reservoir overpressure 0.8 MPa, tube length up to 3 m, separated gas and particles before ejection). They observed a generally collimated jet exiting the cylinder vent, whereas a generally larger spread was observed with converging and diverging vents. Within the converging vent, it appeared that particles were guided by the vent walls in their trajectory. Finally, Cigala et al. (2017) originally performed the experiments that are subjected to re-analyses here, which were designed for a different purpose. They used shock-tube experiments to rapidly decompress gas (argon) and particle mixtures forming transient and underexpanded jets from an initial overpressure in the reservoir of 15 MPa at room and high temperature. They quantified the temporal evolution of particle exit velocity and scaled the dynamics using the isentropic theory for supersonic jets.

In this work, we extend the analysis of Cigala et al. (2017) experiments to constrain the dynamic evolution of the gas and particle spreading angle. We focus on understanding how initial conditions, such as vent geometry or particle load, affect the lateral spreading of gas and particles impulsively ejected into the atmosphere after a rapid decompression event.

## Materials and methods

### Experimental setup and conditions tested

The focus of this study was to reveal through systematic observation the influence of vent geometry, initial tube length, particle load, particle size and temperature on gas and particle spreading angle. Our shock-tube contains a batch of loose volcanic particles at the base and is pressurised using argon (heat capacity ratio *γ*=1.67, specific gas constant *R*=208 J/kg K). Argon is inert, non-toxic and readily available in gas bottles, all factors that outweigh the slight discrepancy of its expansivity compared to those of the major gas species driving explosive volcanic eruptions. Following rapid decompression, the gas expands and accelerates the particles, generating the jets investigated here. The jets are impulsively ejected into a 3.35-m-high tank at ~0.1 MPa, ~25 °C. In all our experiments, the jet characteristics and dynamics have been investigated immediately above the vent (field of view approx. 10×20 cm).

**Fig. 1:**
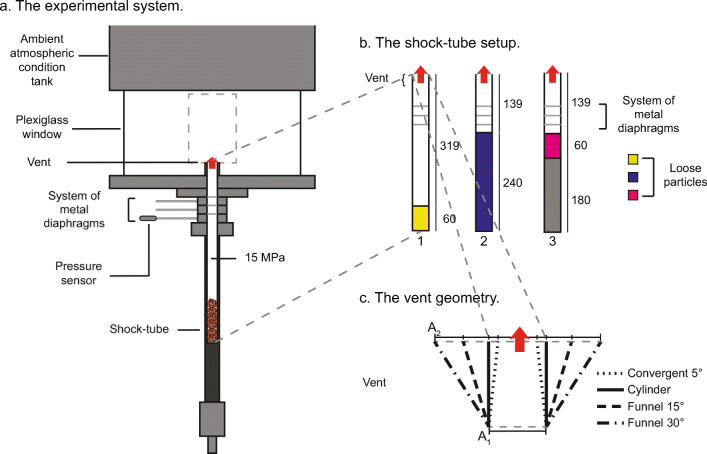
**a** Sketch of the shock-tube system. **b** In yellow, blue, and pink the sample location and volume occupied are highlighted and represent setup 1, 2 and 3, respectively. The same colour code is used in all plots to indicate the setup condition. The grey area in setup 3 represents a full cylinder of Teflon or metal that was only used to reduce the shock-tube volume. **c** Sketch of the different vent geometries used. The area at the lip of the vent (A_2_) changes depending on the vent geometry, while A_1_ is the same for all the geometries. For more details about the experimental setup, see Cigala et al. (2017) and references therein

With the sole exception of ‘setup 1b’ (see below), the experimental conditions tested are those of Cigala et al. (2017) to which a series of gas-only experiments at room temperature has been added. All experiments involving particles were repeated at least three times at each set of conditions, while gas-only experiments were performed only once at each set of conditions. The experimental conditions tested are summarized below as well as in Fig. 1.

1. Vent geometry (Fig. 1c), four different configurations:
  - nozzle with 5° converging walls,
  - cylinder,
  - funnel with 15° diverging walls,
  - funnel with 30° diverging walls.
2. Initial tube length (Fig. 1b, Table 1), defined here as the sample-surface-to-exit distance. (This parameter is not independent of particle load):
  - 319 mm (setup 1),
  - 139 mm (setup 2 and 3).
3. Particles: scoriaceous fragments of a porous basaltic lava flow “Schaumlava”, from the East Eifel volcanic area (Germany). Particle density was 2500 kg/m^3^ and the particle shape parameters are described in Douillet et al. (2014).
  - Particle load (Fig. 1b), considering a loose random packing the void fraction available for the gas within the sample volumes is 0.4, with setup 1 having an ‘extra’ column of gas above the sample surface (in Table 1 the reservoir volume is provided):
    i. 34.8 ± 2.8 g (setup 1),
    ii. 150.9 ± 8.9 g (setup 2),
    iii. 36 ± 3.4 g (setup 3).
  - Particle size, three initial grain size distributions (GSDs), where *d*_*p*_ is estimated via mechanical dry sieving in one *φ* interval:
    i. 1 ≤ *d*_*p*_ < 2 mm (coarse),
    ii. 0.5 ≤ *d*_*p*_ < 1 mm (medium),
    iii. 0.125 ≤ *d*_*p*_ < 0.250 mm (fine).
4. Temperature protocol:
  - Room temperature (RT, ca. 25°C in the figures),
  - High temperature (HT in the figures), particles in the shock-tube were heated to 400–500°C. Theoretical calculations show that initial temperatures within this range give almost identical results.
5. Pressure in the shock-tube (*P*_*r*_): 15 MPa. *P*_*a*_ is the atmospheric pressure (here ca. 0.1 MPa)

**Table 1:** Summary of the setup characteristics, the distance of the sample surface from the vent exit before decompression (in mm), the volume of the sample chamber occupied by particles (in m^3^) and the particle load (in grams). *Averaged over several experiments

Setup	Surface distance	Volume sample chamber	Particle load^*^
	(mm)	(m^3^)	(g)
1	319	3.2×10^-5^	34.8±2.8
2	139	1.3×10^-4^	150.9±8.6
3	139	3.2×10^-5^	36.0±3.4

To analyse the influence of particle load and absolute amount of gas at the start, gas-only experiments were performed for all the vent geometries. In setups equivalent to setup 2 and 3 of the gas-particle experiments (Fig. 1), reservoir volumes of 1.27×10^-4^ and 3.18×10^-5^ m^3^, respectively, were released impulsively at both high and low temperature. As the initial temperature affects the cooling rate and accordingly the condensation-induced gas visibility, optical videos (no schlieren setup was available) of hot and cold initial conditions could not be directly compared in this case.

The high-pressure section of the shock-tube was isolated from ambient pressure via a system of three metal diaphragms (Fig. 1a), with pressure differences across each diaphragm set to 5 MPa, a gas-particle reservoir pressure of 15 MPa was achieved. When the pressure below the top diaphragm was intentionally raised above 5 MPa, this diaphragm failed, leading successively to the failure of the second and third diaphragm and the sample experiencing rapid decompression, generating a gas-particle jet. The diaphragms typically opened with successive delays of 0.1 to 0.3 ms, leading to visually distinguishable pulses of condensed gas. We note that the opening process was sometimes delayed, probably due to material properties of the metal sheets used in fabricating the diaphragms. However, this does not influence the unfolding dynamics once the lowermost diaphragm has failed and the starting jet formed. Here, for the purpose of synchronisation between experiments, time zero was chosen to correspond to the appearance of the third gas pulse. At about 1 – 5 ms (depending on the setup condition), the first particles exit the vent, and particles exit earlier for setups 2 and 3 compared to setup 1.

### Scaling of the experiments

The dynamics of volcanic jets can be characterized by non-dimensional numbers such as the Reynolds number (*Re*), which expresses the ratio of inertial to viscous forces and ranges widely from 10^5^ up to 10^11^ (e.g. Kieffer and Sturtevant 1984; Clarke 2013; Tournigand et al. 2019); and the Mach number (*M*) which expresses the ratio between sound and flow velocity and can range from subsonic, *M* < 1, to supersonic, *M* > 1 (e.g. Kieffer and Sturtevant 1984; Genco et al. 2014; Médici and Waite 2016). The effects of particles and the degree of coupling between gas and particles are expressed by the particle Reynolds (*Re*_*p*_) and the Stokes (*St*) number, respectively. The latter strongly depends on particle size (e.g. Woods 1995; Carcano et al. 2014; Cerminara et al. 2016). In Cigala et al. (2017) *M*, *Re*, *Re*_*p*_ and *St* were constrained by applying the 1D isentropic theory for supersonic jets (Saad 1985, see Appendix A for more details) based on initial (i.e. reservoir) conditions and treating gas and particles as separate phases. Here, we define *P*_*r*_ as the initial pressure in the reservoir and *P*_*a*_ as the atmospheric pressure. Orescanin and Austin (2010) found empirically that underexpanded jets from finite reservoirs with *P*_*r*_/*P*_*a*_ > 15 and a non-dimensional volume > 1 had a similar behaviour to steady jets. This behaviour is even more similar for larger values of both parameters. For the starting conditions investigated here (*P*_*r*_*/P*_*a*_ = 150), the theoretical value of *M* has been estimated using the critical to exit area ratio relationship (see Appendix A for the list of equations used) and found to range from 1 to 3.69 at the vent exit, reaching a theoretical value of 4.39 at the fully expanded conditions downstream, where the flow pressure equals atmospheric pressure. Note that fully expanded conditions do not necessarily represent the maximum *M* within the jet. Furthermore, at these fully expanded conditions, the flow Reynolds number (*Re*) varies between 8.9×10^6^ and 1.3×10^8^, while the particle Reynolds number (*Re*_*p*_) varies from 2.0×10^5^ to 2.3×10^6^, and *St* ranges from 12 to 135 (see Table 3 in Cigala et al. 2017). Accordingly, jets shortly after the release were i) supersonic, ii) underexpanded, iii) in the turbulent regime and iv) particles were not coupled to the gas flow. It is important to stress that the values above are theoretical; the 1D isentropic theory assumes an infinite reservoir and we have considered gas and particles as separate phases, with the result that the dimensionless parameters are likely overestimated. The overestimation may be stronger for the experiments performed with setup 3, which have the smallest reservoir volume. Moreover, as all experiments were triggered from a finite reservoir, the related jets were transient and the related *M*, *Re*, *Re*_*p*_ and *St* evolved dynamically. Finally, the same analysis was not possible for experiments performed with particles 0.125 ≤ *d*_*p*_ < 0.250 mm due to the small size of individual particles and the video resolution making direct measurement of the velocity of single particles not possible (Cigala et al. 2017). Based on the scaling results for the other two GSDs, we assumed the jets to be initially supersonic, underexpanded and in the turbulent regime. For this smaller particle size, *St* will likely approach a value closer to 1, indicative of a possible better coupling with the gas phase.

Upon reaching choking conditions (*M* = 1) and depending on pressure ratio, volume of gas available and vent geometry, the flow may expand further becoming supersonic through expansion fans; the lower pressure and density of the flow makes the flow occupy a larger volume and hence widening. The expansion fans are isentropic and, for a flow going from *P*_*r*_ to *P*_*a*_, their expansion, typically indicated by *ν*(*M*)*,* is given by the Prandtl-Meyer function,

1 $$ \nu (M)=\sqrt{\frac{\gamma +1}{\gamma -1}}{\tan}^{-1}\sqrt{\frac{\gamma -1}{\gamma +1}\left({M}^2-1\right)}{-\tan}^{-1}\sqrt{\left({M}^2-1\right)} $$

where *γ* = 1.67 for argon and *M* is the Mach number associated to that specific expansion. The expansion occurs over a turning angle (*θ*), which is represented by

*θ* = *ν*(*M*_2_) − *ν*(*M*_1_) (2),

where subscripts 1 and 2 represent the flow conditions before and after expansion, respectively.

If the flow undergoes choking, it takes place at the critical area, which in our case corresponds to the vent exit in the converging geometry for example. In this case, *M*_*1*_ = 1 and supersonic expansion only occurs from the exit onwards (*ν*(*M*_*1*_) = 0). Therefore, *θ* = *ν*(*M*_2_) is a measure of the lateral spreading of the jet and can be used to retrieve *M*_*2*_ by resolving Eq.1 inversely.

In contrast, the diverging vents allow the flow to expand as soon as the cross-sectional area is increasing. If, from the exit onwards, the jet expands further, this expansion can be observed as lateral spreading of the jet. However, in order to properly characterize the corresponding *M* using Eq. 1 it is important to correct the measured spreading angle for the vent geometry. For the case under study with a funnel with walls diverging at 15°, we calculated *M* = 2.87 at the vent exit, corresponding to a Prandtl-Meyer expansion *ν*(*M*_*1*_) = 37.03°. The measured jet spreading angle from the vent exit (e.g., 37.1°) corrected for the wall’s divergence (*θ* = 37.1 °  − 15 ° ) leads to *ν*(*M*_2_) = *θ* + *ν*(*M*_1_) = 59.13° which is related to a *M* of 5.4 according to Eq.1. The assumptions above are valid if the gas jet completely filled the vent geometry.

### Video analysis

The gas-particle jets were recorded close to the vent exit with a Phantom (V series) high speed camera at 10,000 fps through a transparent Plexiglass tube. Our videos thereby condense a 3D phenomenon onto a 2D image. Accordingly, the left and right edges of the jet are the extreme edges of a conical gas/gas-particle jet. The argon gas expanded and cooled upon exiting the shock-tube causing visible condensation. We performed image analysis using ImageJ to quantify the evolution of both gas and particle spreading angles with time.

**Fig. 2:**
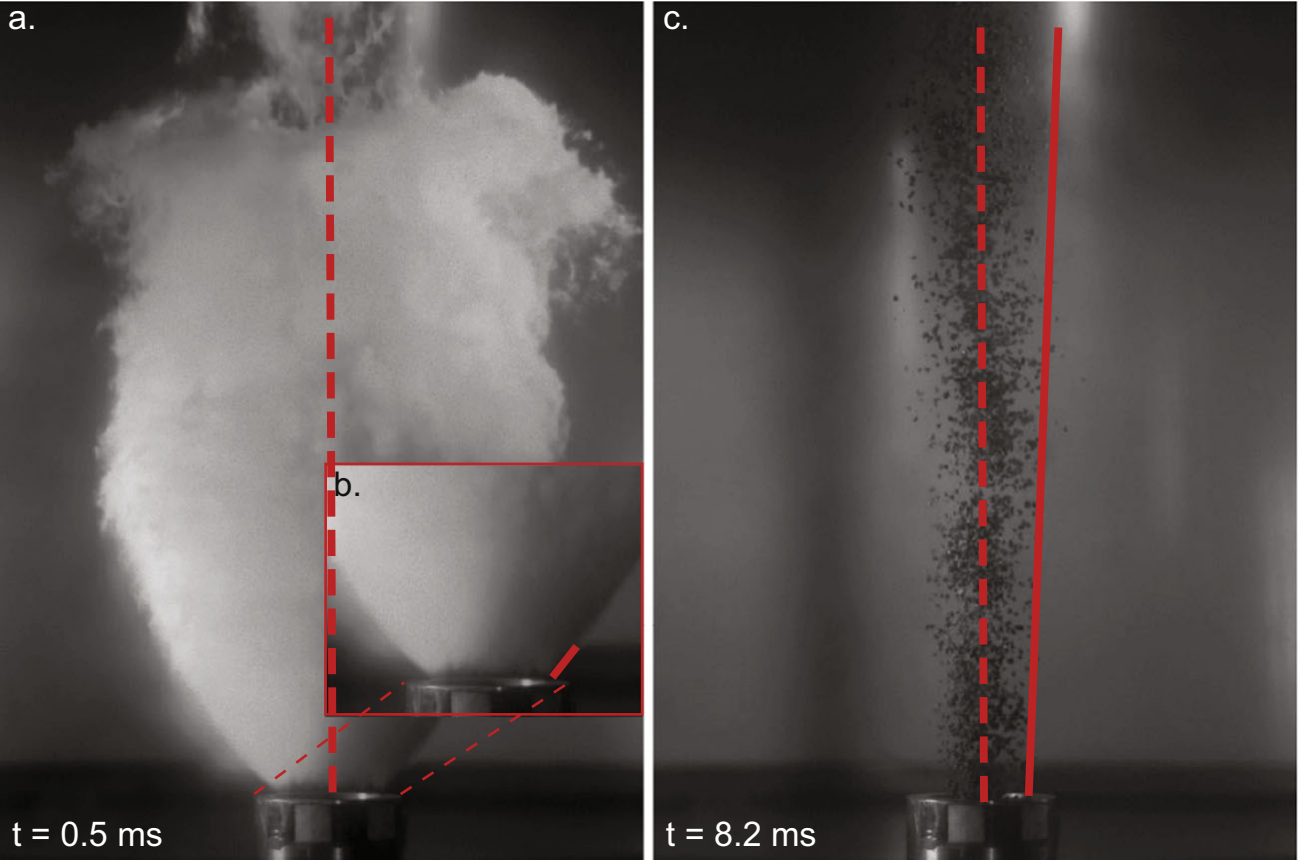
Still frames of high-speed videos used to show how the lateral spreading angle of gas (**a**, **b**) and particle-laden jets (**c**) was derived. The vertical dashed line is the centre line, the vertical trajectory to compare the angular deviation with. The gas spreading angle was measured directly at the vent (**b**). For particles, the entire visible jet length (ca. 20 cm) was considered (**c**). The tangent to particle movement (solid red line) excluded outliers, particles deviating from the main jet because of secondary processes such as particle-particle or particle-wall collision

To measure the gas spreading angle, we drew a short tangent along the visible gas right at the lip of the vent (Fig. 2a and b, for visual representation). All measurements were performed on both sides of the jet and averaged.

To measure the particle spreading angle, we drew a tangent at the edge of the particle-laden jet starting from the lip of the vent and considering the entire vertical field of view (ca. 20 cm, see Fig. 2c for a visual representation). The measurement was taken on both sides and averaged. We conducted the first measurement of the particle spreading angle at the point in time when the jet filled the field of view, which commonly occurs about 0.5 ms after the first particles exit the lip of the vent. The quantification of the particle spreading angle disregarded particles (*n*<10) that showed trajectories deviating substantially from the main particle-laden jet, which were deemed to be outliers. Amongst the likely processes responsible for this erratic behaviour are particle-particle and particle-wall collisions. However, on video, it was not possible to determine which of the processes was responsible. The particle spreading angle was commonly measured until the first falling particles (the ones that had been ejected first) came into sight or until a jet of particles could be objectively recognized (see examples of frame sequences in Fig. S1 and S2 in the Supplementary Material).

The uncertainty in the measurements is described by the standard deviation on left- and the right-side measurements between repeated experimental conditions. In general, the standard deviation is smaller for particle spreading angle than for gas spreading angle. The largest uncertainty (up to 5.9° on maximum gas spreading angle before particle exit) was found for the gas spreading angle measurements from high temperature experiments due to less condensation of the gas during expansion. This makes it impossible to determine the gas spreading angle in hot, gas-only experiments and makes the estimation of the gas spreading angle in hot experiments with particles nontrivial. This is particularly true for setup 1 experiments, where the larger gas volume on top of the particles condenses much less upon exit than in setup 2 and 3. In addition, we tested the objectivity of the operator by asking two independent operators to determine the gas and particle spreading angle on four frames where gas was present and four frames where particles were present. Among operators, the maximum deviation is 2.8° on gas spreading angle, and 0.8° on particle spreading angle. The measurements have a low operator subjectivity.

From these measurements we evaluated:

1. The maximum gas spreading angle before particles exit the vent, reflecting the maximum lateral expansion the gas undergoes prior to visible influence due to particles. The latter does not imply that the presence of particles in the shock-tube does not influence initial gas dynamics.
2. The initial particle spreading angle, reflecting the lateral divergence of the particle-laden jet at the initial point in time when it fills the entire field of view above the lip of the vent.
3. The evolution of both gas and particle spreading angle with time.

## Results

### Maximum gas spreading angle

**Fig. 3:**
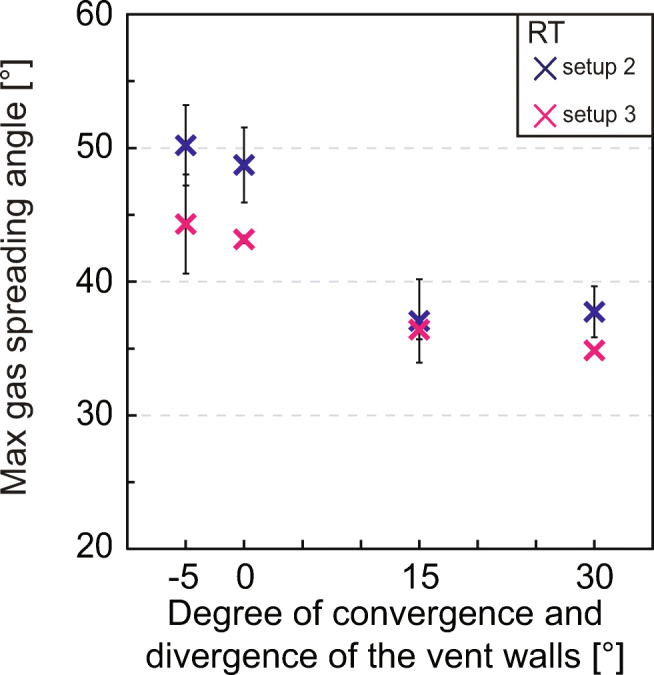
Maximum gas spreading angle at exit plotted against the vent geometry represented by the degree of convergence and divergence of the vent walls for gas-only experiments performed at room temperature. -5 = nozzle with 5° converging walls, 0 = cylinder, 15 = funnel with 15° diverging walls, 30 = funnel with 30° diverging walls. Each point and associated error bar represent the average and range of the maximum gas spreading angle measured on both sides of the gas. These experiments were not repeated. Error bars can be smaller than the related symbol

**Fig. 4:**
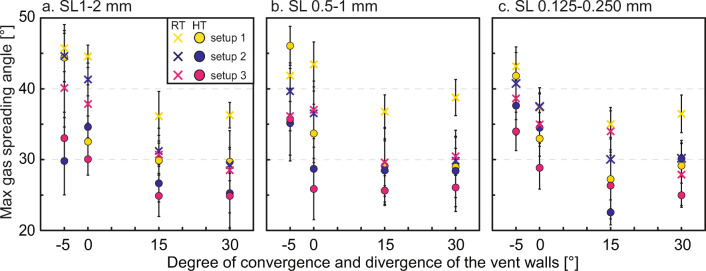
Maximum gas spreading angle at the exit, before the first particles exit the event, plotted against the vent geometry represented by the degree of convergence and divergence of the vent walls. -5 = nozzle with 5° converging walls, 0 = cylinder, 15 = funnel with 15° diverging walls, 30 = funnel with 30° diverging walls for particles with a diameter of a) 1 ≤ *d*_*p*_ < 2 mm, b) 0.5 ≤ *d*_*p*_ < 1 mm, c) 0.125 ≤ *d*_*p*_ < 0.250 mm, respectively. Each point and relative error bar represent the average and standard deviation of the gas spreading angle measured on both sides of the gas jets of at least three repetitions (i.e. at least six measures per angle) at the same initial conditions. Dots are for hot experiments, crosses for room temperature. Error bars can be smaller than the related symbol

The maximum gas spreading angle for gas-only experiments performed at room temperature (RT) is shown in Fig. 3. For particle-gas experiments, Fig. 4 represents the maximum spreading angle measured before particles exit the vent. The results are shown as a function of the degree of divergence of the vent walls and, for the experiments with particles, split according to the initial GSD (see Material and Methods for details) in coarse (Fig. 4a), medium (Fig. 4b) and fine (Fig. 4c). In all experiments, the nozzle with converging walls caused the largest changes in spreading angle, peaking at 50.2±3° (gas-only experiments) and 46.1±2.7° (gas-particle experiments). Maximum gas spreading angle is typically negatively correlated with temperature. In setup 1 experiments (Fig. 4), where the sample surface-to-exit distance is larger and particles exit the vent later than setup 2, the maximum gas spreading angle shows larger values than setup 2 and setup 3. Indeed, gas-only experiments also show maximum values larger for setup 2 than for setup 3, likely due to the smaller volume of gas ejected in the latter.

**Fig. 5:**
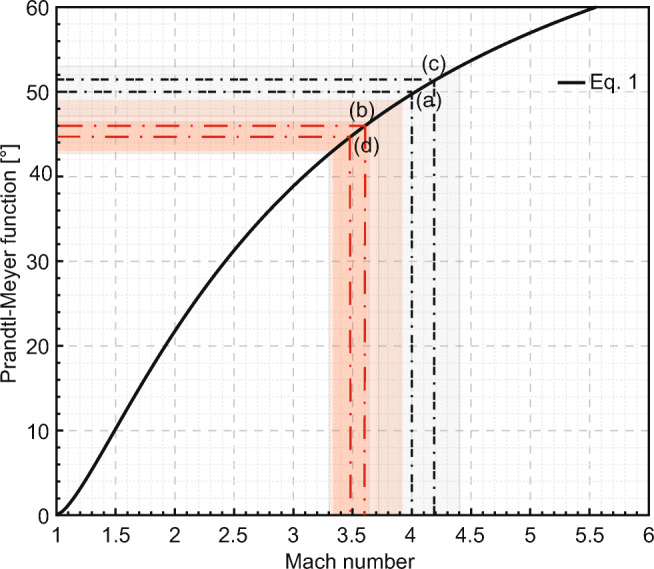
Prandtl-Meyer expansion function versus Mach number as estimated from Eqs. 1 and 2. Points (a) and (b) represent the expansion function for the maximum gas spreading angle measured in experiments with gas-only and gas and particles, respectively. Points (c) and (d) represent the expansion function for the minimum gas spreading angle measured in gas-only and gas and particles experiments, respectively

We used the values of maximum gas spreading angle to retrieve *M* according to Eqs. 1 and 2 (Fig. 5). For gas-only experiments and the converging vent geometry, the spreading angle reaches a value of 50.2±3° corresponding to 3.72 ≤ *M* ≤ 4.43 (Fig. 5, point a). In experiments with particles and converging vent geometry, the spreading angle reaches 46.1±2.7°, corresponding to 3.37 ≤ *M* ≤ 3.9 (Fig. 5, point b). In experiments with positively diverging vent geometries, the measured spreading angle is commonly lower down to 34.8±0.1° (gas-only and funnel with 30° diverging walls) and 22.6±1.8° (gas-particle and funnel with 15° diverging walls). After wall divergence correction, they correspond to *ν*(*M*) 51.6±0.1° (Fig. 5, point c) and 44.6±1.8° (Fig. 5, point d), respectively. Accordingly, we obtain 4.18 ≤ *M* ≤ 4.2 for the gas-only and funnel with 30° diverging walls, and we obtain 3.31 ≤ *M* ≤ 3.64 for the gas-particle and funnel with 15° diverging walls.

### Initial particle spreading angle

**Fig. 6:**
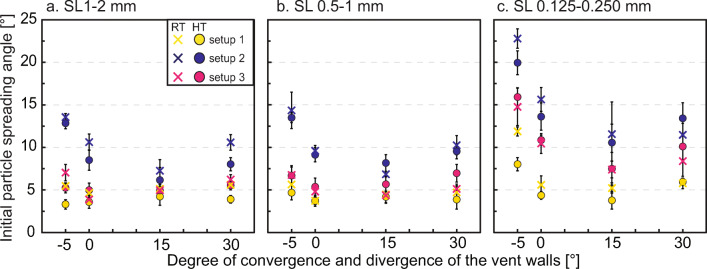
Initial particle spreading angle plotted against vent geometry represented by the degree of convergence and divergence of the vent walls. -5 = vent with 5° converging walls, 0 = cylinder, 15 = funnel with 15° diverging walls, 30 = funnel with 30° diverging walls for particles with a diameter of a) 1 ≤ *d*_*p*_ < 2 mm, b) 0.5 ≤ *d*_*p*_ < 1 mm, c) 0.125 ≤ *d*_*p*_ < 0.250 mm, respectively. Each point and relative error bar represent the average and standard deviation of the particle spreading angle measured on both sides of the particle jets of at least three repetitions (i.e. at least six measures per angle) at the same initial conditions. Dots are for hot experiments, crosses for room temperature. Error bars can be smaller than the related symbol

Considering the different experimental conditions and with the exception of experiments with converging vent geometry and setup 2, coarse (Fig. 6a) and medium (Fig. 6b) particles show comparable values of initial particle spreading angle, typically below 10°. In experiments with fine particles (Fig. 6c), initial particle spreading angles of up to 22.8±1.4° are observed. Initial particle spreading angles consistently show smaller values than the corresponding gas spreading angle at experiment start and are most noticeably affected and positively correlated with particle load.

Setup 2 commonly displays the largest spreading angle, while setups 1 and 3 show similar results, with only 1–3° larger spreading angles for setup 3 experiments using coarse and medium particles. However, with fine particles, the initial particle spreading angle has been observed to be 4–10° larger in setup 3 experiments compared to setup 1 experiments. We argue here that the latter finding implies that the tube length plays a significant role in the setup 3 experiments with fine particles.

Vent geometry, in general, exerts a smaller control on particle spreading angle compared to particle load. By analogy to the spreading behaviour of the gas, the vent with converging walls commonly shows the largest initial particle spreading angles values (up to 23°), while the funnel with 15° diverging walls the smallest ones (< 13° for setup 2). The cylinder and the funnel with 30° diverging walls show more similar trends for all conditions. The temperature shows a minor control on the initial particle spreading angle. Only in experiments using the converging vent geometry and fine particles in setups 1 and 2, could a difference of more than 3° be measured.

### Temporal evolution of gas and particle spreading angle

**Fig. 7:**
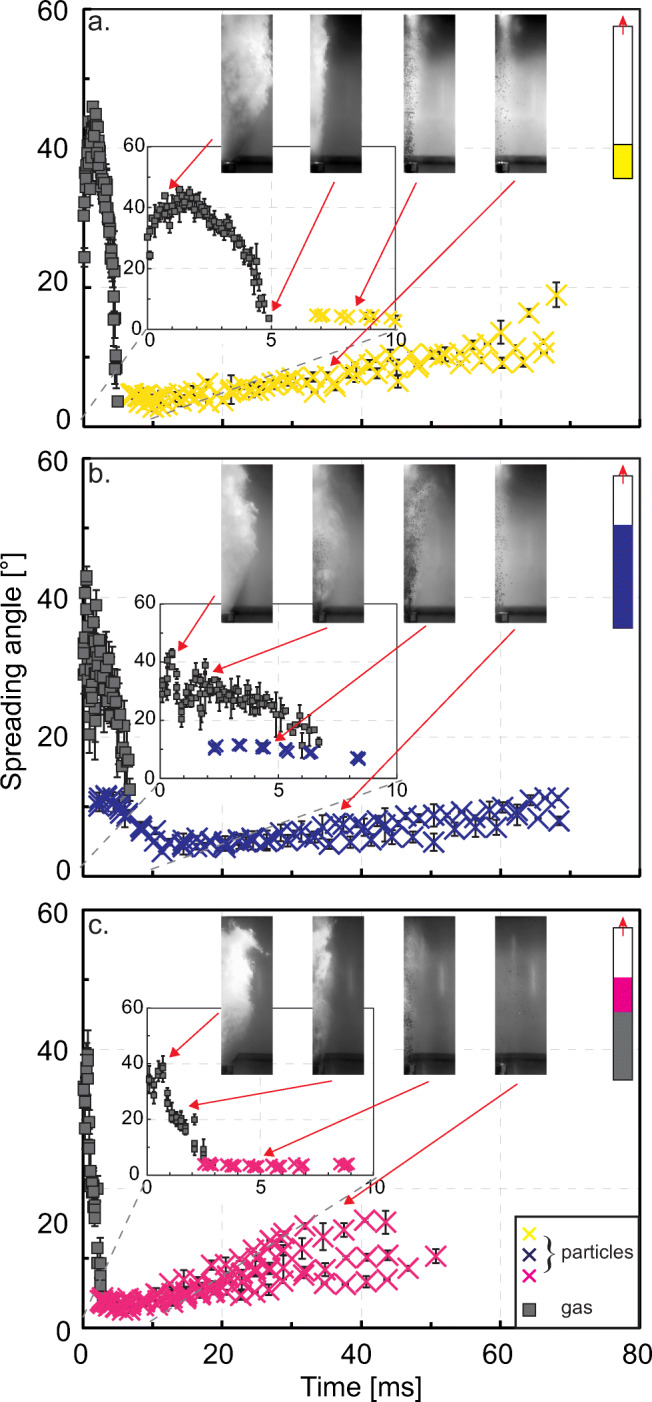
Temporal evolution of the spreading angle of gas (grey square) and particles (cross) for constant particle size (SL 1–2 mm), initial overpressure (15 MPa), initial temperature (25°C), and geometry (cylindrical vent). **a** setup 1, **b** setup 2 and **c** setup 3 as sketched in the inset, the coloured part corresponds to sample volume in the tube. Frames from the videos (approximately 25 cm high and 8 cm wide, vertically cut along the centre line) indicate gas and particle behaviour, the red arrows correspond to the still frame acquisition time. A more complete frame sequence for the conditions shown here is available in Fig. S1, Supplementary Material. The gas spreading angle evolves in a bell shape trend for the long conduit (**a**). The short delay between gas and particle arrival indicates the impact of particles on gas expansion and originates a secondary peak (**b**). This effect is visible but less pronounced in (**c**). Time zero is set at the appearance of the third pulse of gas (from the lower diaphragm opening) in the video, the delay of particle exit is positively correlated with tube length (the distance of sample surface from vent exit) before decompression. Each point and relative error bar represent the average and range of the gas and particle spreading angle measured on both sides of the jets. Error bars can be smaller than the related symbol. Maximum gas spreading angle is similar but with temporal variability. After the initial spreading, the particle-laden jets can collimate, around approx. 10 ms, and later they flare for all experimental conditions

**Fig. 8:**
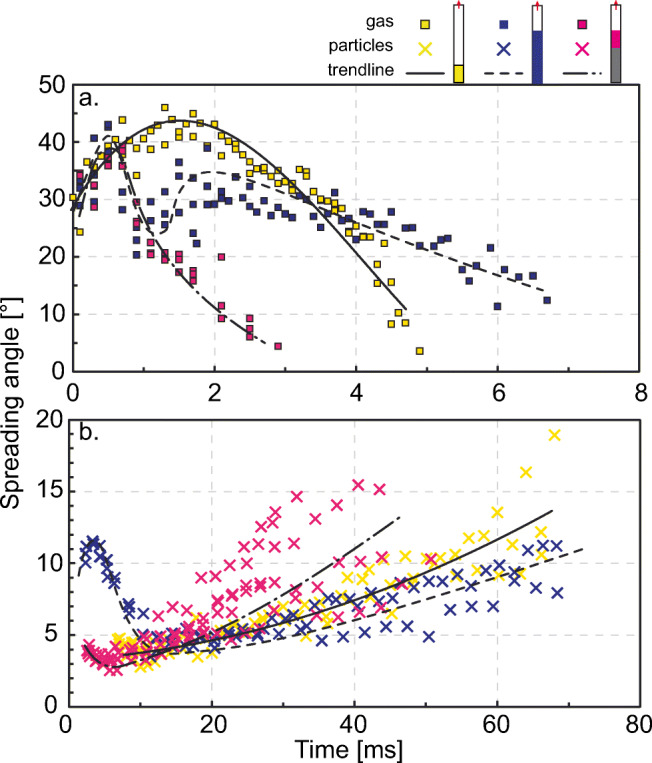
Same trends of gas and particle spreading angle as presented in Fig. 7 but plotted to compare setups. In this case, also the gas squares are represented in with the setups colour code

Figure 7 shows an example of spreading angle evolution for both gas and particles for experiments performed at room temperature, with the cylinder vent geometry, coarse particle fraction and setup 1 (Fig. 7a), setup 2 (Fig. 7b), and setup 3 (Fig. 7c), respectively. The plots were compiled from three repeat experiments, and the error bars represent the deviation between angle measurements taken on the right and the left side of the jets. At comparable times, the maximum variation between angles is between different repetitions. To further illustrate the differences between the three setups, we compare the trends of Fig. 7 in Fig. 8. The temporal evolution of the gas and particle spreading angle for all the different experimental conditions tested are plotted in Fig. S3-S8 and can be found in the Supplementary Material. Examples of frame sequences are also available in Fig. S1 and S2 in the Supplementary Material. In Fig. S9 (Supplementary Material), similarly to Fig. 8, we present the overlapping of the temporal evolution of the gas spreading angle for gas-only compared to gas and particle experiments.

The gas spreading angle evolution (Fig. 7, grey squares) is best illustrated for setup 1 (Fig. 7a) where it shows a peaked evolution. In this case, the gas jet is least perturbed by particles, which are ejected up to 5 ms later than in setup 2 and 3 and clearly shows the initial expansion and subsequent narrowing (see also Fig. 8a). In the case of setup 2 and 3, the gas is still visible when the first particles are ejected from the vent and likely disturb gas dynamics. In the latter cases, the arrival of particles corresponds in some instances to a further lateral expansion of the gas, shown in the graphs as a secondary peak (e.g. Fig. 7b, 9d). The values of the gas spreading angle whilst particles are also exiting can show larger deviations from the left to the right side of the jet indicating a more complex adjustment to lateral expansion due to the presence of particles.

**Fig. 9:**
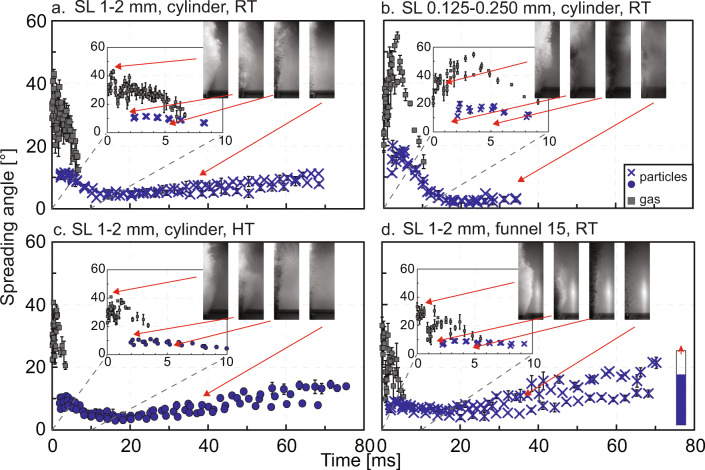
Comparison of the temporal evolution of gas and particle spreading angle for experiments performed with setup 2 and **a** coarse particle fraction, room temperature, cylindrical vent, **b** fine particle fraction, room temperature and cylinder, **c** coarse particle fraction, hot temperature, cylinder, and **d** coarse particle fraction, room temperature and funnel with walls diverging at 15°. Time zero is set at the appearance of the third pulse of gas in the video, particles follow the gas after a variable delay. See also Fig. S1 and S2 in Supplementary Material. Dots are for hot experiments, crosses for room temperature experiments. Each point and relative error bar represent the average and range of the gas and particle spreading angle measured on both sides of the jets. Error bars can be smaller than the related symbol. Four still frames are also plotted for each experimental condition; their time is indicated by the red arrows. The frames are cut in half to fit more frames in one figure

The temporal evolution of particle spreading angle differs sharply between the three setups. Setup 1 (Fig. 7a and 8b, and Fig. S1 and S2, upper sequence) commonly shows the smallest initial spreading angles, with particles following an almost vertical trajectory, and a subsequent steady increase. A larger initial spreading is displayed only in experiments performed with the converging vent geometry and the fine particle fraction (Fig. S7a and S8a). After about 20 ms, in most experiments, we observe that an increasing number of particles follow non-vertical trajectories. Setup 2 (Figs. 7b and 8b, and Fig. S1 and S2, middle sequence) frequently shows an increase towards a first peak (after about 3 ms). Later, the jet tends to collimate towards a quasi-vertical ejection (like in setup 1 experiments), followed by a final increase of the radial spreading after about 20–30 ms from ejection onset. Setup 3 (Figs. 7c and 8b, and Fig. S1 and S2, bottom sequence) follows an intermediate path. At the onset, the spreading angle is initially 1–10° larger than in setup 1 experiments, but 2–7° smaller compared to setup 2. It sometimes shows an initial increase towards a peak, followed by the ‘typical’ quasi-vertical pattern and the final radial spreading from around 20 ms onwards. Setup 3 experiments also show an early end of the ejection, with a few particles exiting the vent at ca. 40 ms, given the smaller volume involved, or with particle falling back and obscuring the image after 20–30 ms. Early fall back of particles is more common for experiments with fine particles in setup 2. See also the bottom sequence of Fig. S1, and plots in Fig. S7 and S8 in Supplementary Material for further comparisons.

Figure 9 shows the temporal evolution of the gas and particle spreading angle as a function of different experimental conditions, namely particle size (Fig. 9a and b), temperature (Fig. 9a and c), and vent geometry (Fig. 9a and d). In the case of vent geometry, data for the funnel vent with diverging walls at 15° (Fig. 9d) show smaller peak values for gas and particle spreading angle, as well as a greater range in the particle spreading angle starting from ca. 35 ms, compared to the cylinder case (Fig. 9a). At otherwise constant conditions, vent geometry leads to 1) a change in the maximum initial value for the angle of both gas and particles (see also Figs. 3 and 4), and 2) a larger (for diverging geometries) or smaller (for the converging geometry) lateral deviation of the particles around 30–60 ms. The temperature shows commonly a minor influence, and it is limited to gas and particle ejection onset (Fig. 9a and c). A smaller particle size commonly shows a larger initial particle spreading angle value (Fig. 9a and b). As also mentioned in the paragraph above, experiments performed with the fine fraction may result in shorter durations compared with experiments performed with the coarser particle fraction.

In experiments with converging and cylindrical vent geometry, the particle-laden jets initially occupied the entire cross-sectional vent area. In other words, the jet diameter at ejection onset was always as large as the vent exit diameter (23 and 28 mm for nozzle and cylinder, respectively). This implies that the gas-particle jet must pass through a bottleneck, in particular within the converging vent, undergoing collimation (increased particle number density) and hence acceleration, thus affecting the decompression rate at the lip of the vent, but also causing more particle-particle and particle-walls interactions. In contrast, when particles were ejected from the vents with diverging walls, the initial jet diameter was larger than 28 mm (the value of every vent diameter at A_1_, Fig. 1), but commonly smaller than the vent exit diameter of 46 and 53 mm (the value of vent diameter at A_2_, Fig. 1), respectively for vent walls diverging at 15° and 30°. At the onset of setup 2 experiments with diverging geometry and using fine particles, the initial jet diameter was as large as the vent exit diameter.

The converging vent geometry seems to promote spreading at the ejection onset, whereas the effect is inverted toward the end of the ejection (from around 40 ms on) and the few slow particles still being ejected are inherently less laterally deviated than in experiments with diverging geometries. Once particles have lost most of their momentum, a particle exiting from the diverging vents with an outward deviating trajectory will not find obstacles to its path. In contrast, the results show that in the converging geometry, precisely because the exit is narrower, particles might interact with the walls before exit, rebound and set out slightly more vertical.

## Discussion

Although laboratory experiments are smaller and of shorter duration than explosive volcanic eruptions, they share some of the same process dynamics (e.g. Kieffer and Sturtevant 1984; Chojnicki et al. 2006; Cigala et al. 2017). Acoustic analysis of gas-only experiments using the same setup (Peña-Fernández et al. 2020) revealed that most transient jets are composed of three stages, 1) an early vortex head phase, 2) a quasi-static phase and 3) a trailing phase, depending on pressure ratio and reservoir volume. Thus, by scaling experiments and volcanic eruption non-dimensionally, it is possible to infer from the former which variables are likely influencing the latter. Due to the thermal disequilibrium between gas and particles in our experiments, we preferred the assumptions of the 1D isentropic theory over the pseudogas approximation and scaled the experiments non-dimensionally as supersonic jets, considering gas and particles as separate phases. We are aware that this assumption may lead to an overestimation of the initial gas velocity. However, the pseudogas approximation has also led to under- and over-estimations of observables in previous experimental investigations because of the lack of adequate interphase drag terms (Chojnicki et al. 2006; Alatorre-Ibargüengoitia et al. 2010). The pseudogas approximation equations proposed by Alatorre-Ibargüengoitia et al. (2010), who used the same shock-tube apparatus and similar experimental conditions, underestimated measured particle velocities by Cigala et al. (2017) by up to 50%.

### Gas spreading angle

The measured maximum gas spreading angle can be used to resolve the Prandtl-Meyer expansion function (Eq. 1) to provide an estimation of the related *M*. In experiments with particles, the maximum spreading angle was smaller, showing that the presence of particles affects gas dynamics mainly by reducing the initial gas volume and increasing energy consumption due to particle acceleration. Orescanin and Austin (2010) found that for pressure ratios > 15, underexpanded jets from finite reservoirs with a non-dimensional volume > 1, show characteristics of those from infinite reservoirs. These characteristics are even more similar for larger values of the two parameters. In our study, the initial pressure ratio was always 150 and the rapid decay was modulated by the vent geometry as well as the initial gas volume available (smallest in setup 3). The earlier ejection of particles in setups 2 and 3 (shorter initial sample-surface-to-exit distance compared to setup 1) also affects the expansion dynamics of the gas jet. The presence of large particles or particle clusters affects the flow of gas inside and above the shock-tube and may lead to local “extra” lateral spreading, visible as apparent larger-than-theoretically-calculated gas spreading angles (secondary peak in gas spreading angle in Fig. 9b). Such phenomena have not been considered when constraining the maximum gas spreading angle.

We observed a negative correlation between gas spreading angle and temperature in agreement with the positive correlation of gas density and gas spreading angle found by Kieffer and Sturtevant (1984) from shock-tube experiments using light (Helium and Nitrogen, 4 and 28 g/mol, respectively) and dense gases (Freon 12 and Freon 22, 121 and 86.5 g/mol, respectively). RT and HT experiments reveal very similar time delay between gas and particle ejection at otherwise identical conditions. We interpret this as indicating generally incomplete coupling between gas and particles.

The temporal evolution of gas spreading angle agrees with theoretical consideration of reservoir discharging by Kieffer and Sturtevant (1984) and is related to overpressure at the vent and accordingly to *M.* With the topography known and near-vent gas dynamics observed, the same estimation can be retrieved from volcanic eruptions. It would be of interest to combine the estimation from spreading angle with those retrieved via acoustic analysis (Lacanna and Ripepe 2020).

### Particle spreading angle

When particles exit the vent, the pressure in the reservoir has decreased since the start of the decompression, resulting in a difference of overpressure at the vent. Both the initial values and the temporal evolution of particle spreading angle show a link with overpressure at the vent. We note that, in contrast with our results, Sommerfeld (1994, Fig. 10a–c) presented a slightly negative correlation between particle spreading angle and initial reservoir-to-ambient pressure ratios. It remains unclear if their finding was observed during later stages of the gas-particle jets compared to our observational time window. Moreover, they also observed a negative correlation between particle number density at exit and particle velocity. This is in contrast with findings by Cigala et al. (2017), if we assume that a larger initial particle load will lead to larger values of the particle number density at exit. Additionally, in Cigala et al. (2017) and in our study, particle load and sample-surface-to-exit distance were not independent variables, which complicates further comparisons with the Sommerfeld experiments.

The limited spreading in setup 1 (Figs. 1 and 6) is in part due to dissipation of gas overpressure inside the conduit before leaving the vent. We observed that experiments performed with setup 2 (Figs. 1 and 6) showed larger initial values of particle spreading angle and larger peaks after onset. In setup 2, the total volume of gas in contact with the loose pack of particles is larger than in the other two setup configurations. We argue that enhanced permeable gas flow through the non-cohesive, expanding particle mixture contributes to overpressure at the vent, especially during the exit of the first particles, and favours enhanced lateral drag. Future work should aim at further addressing the role of particle bed permeability on gas flow percolation. Johnson et al. (2020) performed laboratory experiments employing a passive gas tracer and showed a positive correlation between particle size and gas percolation velocity just above the particle bed. Applying this result to our system, it may in part help explaining the smaller particle spreading angle observed in coarse particle compared to fine particle jets. An increased percolating gas velocity may reduce gas overpressure and related lateral drag when particles exit. In addition, fine particles, which are particles with typically low *St,* could more rapidly follow the lateral gas expansion and gas turbulence (Sommerfeld 1994; Carcano et al. 2014).

Particle-wall and particle-particle interactions, not limited to particle-particle collisions, may contribute to the spreading angle of particles as electric discharges have been observed in these and similar experiments (e.g. Cimarelli et al. 2014; Méndez Harper et al. 2018; Gaudin and Cimarelli 2019). We did not observe any particle-particle collisions in the videos. A few particles were observed to divert from the main jet trajectory likely because of a strong collision with other particles or the setup before exit. Collisions between particles and vent walls are likely more numerous for the converging and cylindrical geometries than for the diverging ones.

### Volcanic eruptions

Can the experimentally constrained empirical relationships be translated to a better understanding of explosive eruptions? In volcanic eruptions, two kinds of particles can be ejected, pyroclasts generated during this explosive event and “older” clasts that are lying on top of the magma column. The latter ones (typically non-cohesive) are accelerated by the eruption jet from below. Depending on size, they will follow ballistic trajectories from a quasi-static point of departure or may be entrained within the plume. The pyroclasts have been generated by fragmentation processes due to a combination of i) gas overpressure, ii) magma acceleration, iii) magma surface tension and iv) conduit/vent geometry (open vents). Assuming non-homogeneous deformation within the conduit, magma failure is initiated where deformation is highest and stress localised. Magma ascent velocity and the rate at which magma properties (volatile content, porosity and permeability) change, as well as the excavation rate during explosive eruptions, affect the depth of the free magma surface. Accordingly, the depth inside the conduit from which new pyroclasts can be formed and ejected is strongly dynamic. Pulsations in pyroclast velocity have been observed during several eruptions. For example, at Stromboli, such patterns have been correlated with possible complex plumbing system characteristics, related slug ascent scenarios and variable crater and vent area morphologies and their interplay (Taddeucci et al. 2013; Salvatore et al. 2018). It remains speculative if this observation is due to the burst of a train of slugs or the burst of smaller, non-conduit filling gas pockets that may rise quasi-simultaneously. Accordingly, reliably deciphering free magma surface and conduit/vent geometry is difficult and still affects estimations in natural eruptions with large uncertainty (e.g. Gaudin et al. 2014; Dürig et al. 2015; Salvatore et al. 2018; Tsunematsu et al. 2019). Our scaled experiments demonstrated an empirical correlation between initial conditions and gas and particle spreading angle resulting in a complex interplay between 1) particle inertia, 2) particle load, 3) vent geometry (gas expansion dynamics) and 4) effective overpressure at the vent. Future high-resolution observations shall target the dynamics of gas and particle dynamics (lapilli and smaller) in order to reveal if observed trajectories are primarily due to gas overpressure or the effect of ground acceleration shortly before the explosion. Without lateral confinement, trajectories could vary from the vertical path (0°) by 90° to all directions. We believe that a holistic investigation of explosive eruptions, including high-resolution topography with near-vent observation and monitoring data (such as pyroclast content in jets, eruption duration, acoustic signature) will allow a quantitative mechanistic description of the underlying or initial conditions of explosive volcanic eruptions. Near-vent observations of jets dynamics are safer at active mildly Strombolian volcanoes than during more energetic, Vulcanian or Plinian, eruptions. However, we ask for deployment of visual monitoring systems (high-resolution, highspeed) at safe distance (with strong zoom) also at volcanoes in unrest where the explosivity is likely higher than at Stromboli and investigate the initial stages of eruption jets before visibility is expected to be obscured by billowing ash clouds. Albeit at lower temporal resolution than the highspeed cameras used in our laboratory study, increasingly powerful (in frames per second) and low energy consumption cameras, deployed at prominent observation points in sturdy containers or little concrete bunkers, are a great opportunity to capture the jet dynamics and analyse upon eruption end. Flight regulations and distance permitting, Uncrewed Aerial Vehicles (UAVs) will continue their important role of gaining additional observational and analytical information of eruption dynamics and parameters (e.g. James et al. 2020; Liu et al. 2020; Schmid et al. 2021).

## Conclusions

Volcano monitoring cannot yet peer into volcanic conduits to directly observe the conditions underlying an explosion. We aimed here to bridge the lack of direct observations of processes in the uppermost part of volcanic conduits and the consequent lack of understanding of eruptive behaviour by a series of scaled and repeatable laboratory experiments. Rapid decompression experiments of a shock-tube at 15 MPa generated gas jets with mixtures of non-coupled (*St* >> 1) to possibly better coupled (*St* ≈ 1) particles. We investigated the temporal dynamics of gas and particle spreading angle in the near-vent region.

The gas spreading angle evolved quickly towards a peak before it decreased. The pattern was especially appreciable in gas-only and setup 1 experiments, due to the lack, or comparatively late arrival, of particles. For the experimental conditions tested, gas spreading angle showed the following relationships, in order of importance:

1. A negative correlation with vent geometry (larger angle for smaller exit area).
2. A negative correlation with temperature (gas density).
3. A positive correlation with tube length (larger volume of gas).
4. A positive correlation with particle size (higher sample permeability) and negative correlation with particle load (less free gas and loss of potential energy for particle acceleration).

In order of importance, the initial particle spreading angle showed:

1. A positive correlation with particle load (higher probability of lateral rebounding effects or reduced gas permeability).
2. A negative correlation with particle size, with a non-linear effect of the finest particle fraction (better coupling with the gas phase).
3. A negative correlation with vent critical area ratio (small effect).
4. A negative correlation with temperature (small effect).

The particle spreading angle evolution showed patterns varying with particle load and tube length. The vent geometry affects mainly the initial particle spreading angle, its maximum values, enhanced in experiments with the converging geometry, and the final deviations, enhanced in experiments with diverging geometry. Experiments performed with coarse and medium particle size showed remarkably similar evolutions, while experiments performed with the fine fraction showed a similar trend, but much more enhanced in terms of initial maximum values and later evolution. The results presented reveal partly the capability of gas expansion on particle spreading angle, but also the active role of particle-particle and particle-wall interaction. The efficiency of deflection, however, is dependent on i) overpressure at the vent and ii) grain size.

We advocate that attention should be given to high-resolution investigation of jet dynamics in the gas-thrust region directly above the vent as the jet dynamics allow for conclusions about vent geometry and/or gas overpressure at the vent.

## Appendix A

We report here the equations from the isentropic theory for supersonic jets (Saad 1985) applied to estimate gas velocity and properties and the equations used for the non-dimensional scaling of the experiments at initial conditions by Cigala et al. (2017). The area ratio between exit (*A*_*2*_, Fig. 1) and critical area (A*), which is the smallest area the flow has to pass through, was resolved to estimate the Mach number (*M*) at the lip of the vent as follows:

$$ \left(\frac{A_2}{A^{\ast }}\right)=\left({\left(\frac{2}{\gamma +1}\right)}^{\frac{\gamma +1}{2\left(\gamma -1\right)}}\right)\frac{1}{M}{\left[1+\left(\frac{\gamma -1}{2}{M}^2\right)\right]}^{\frac{\gamma +1}{2\left(\gamma -1\right)}} $$(3).

where *γ* is the gas heat capacity ratio. For the present experimental system, A* corresponds to the exit area for the vent with converging walls and to the area of the diaphragms (tube diameter here varies between 26 and 28mm because of how the diaphragms open) for all the other geometries. The *M* values obtained are the so-called designed *M* and their actual applicability further depends on the pressure ratio between the reservoir (*P*_*r*_) and the external ambient pressure (*P*_*a*_). This relationship is expressed as follows:

$$ \frac{P_r}{P_a}={\left(1+\frac{\left(\gamma -1\right){M}^2}{2}\right)}^{\frac{\gamma }{\gamma -1}} $$ (4).

The flow Reynolds number (*Re*) defines the ratio of inertial to viscous forces in a flow:

$$ \mathit{\operatorname{Re}}=\frac{\rho UL}{\mu } $$ (Eq. 5),

where *ρ* and *μ* are the fluid density and dynamic viscosity respectively, *U* the flow velocity and *L* a characteristic length, for example, the vent radius.

The Stokes number (*St*) describes the particle inertial response to the flow, and it is calculated as follows:

$$ St=\frac{\tau_pU}{L} $$ (6)**,**

where *τ*_*p*_ is the characteristic relaxation time of the particles and it is calculated from equation (Elghobashi and Trusdell 1993; Carcano et al. 2013):

$$ {\uptau}_{\mathrm{p}}=\frac{\uprho_{\mathrm{p}}{\mathrm{d}}_{\mathrm{p}}^2}{0.33{\operatorname{Re}}_{\mathrm{p}}\upmu} $$ (7),

where *ρ*_*p*_ is the particle density, *d*_*p*_ is the particle diameter, *μ* is the fluid dynamic viscosity and *Re*_*p*_ is the particle Reynolds number. *Re*_*p*_ serves as correction factor accounting for relative velocities between gas and particles. *Re*_*p*_ is calculated according to the following equation:

$$ {\operatorname{Re}}_{\mathrm{p}}\frac{{\mathrm{d}}_{\mathrm{p}}\uprho \left|\mathrm{U}-{\mathrm{u}}_{\mathrm{p}}\right|}{\upmu} $$ (8),

where *u*_*p*_ is taken as the maximum measured particle velocity at beginning of particle ejection and *U* is the flow velocity theoretically estimated using the following equation:

$$ U={\left[\frac{2\gamma R{T}_0}{\gamma -1}\left(1-{\left(\frac{P_{\infty }}{P_0}\right)}^{\frac{\gamma -1}{\gamma }}\right)\right]}^{1/2} $$ (9),

where *T* is temperature and *P* pressure. The subscript 0 indicates initial conditions in the tube before decompression, while subscript ∞ indicates ambient conditions and *R* is the specific gas constant.

## Supplementary information

ESM 1 (DOCX 32 kb)

ESM 2 (EPS 83339 kb)

ESM 3 (EPS 86023 kb)

ESM 4 (EPS 6089 kb)

ESM 5 (EPS 5377 kb)

ESM 6 (EPS 6136 kb)

ESM 7 (EPS 5365 kb)

ESM 8 (EPS 5700 kb)

ESM 9 (EPS 5718 kb)

ESM 10 (EPS 1743 kb)

## Data Availability

The list of experiments performed are included in a table in the supplementary material and the spreading angle raw data has been deposited in the GFZ Data Services repository (Cigala et al. 2021). The original high-speed videos are archived in the LMU repository and can be accessed upon request.
