# Supplementary material for: Linking gas and particle ejection dynamics to boundary conditions in scaled shock-tube experiments
Source: Bull Volcanol. 2021 Jul 20;83(8):53. doi: 10.1007/s00445-021-01473-0 (PMC8550467; doi:10.1007/s00445-021-01473-0)
Supplement: Supplementary file 1 — (DOCX 32 kb) [file 445_2021_1473_MOESM1_ESM.docx]

# Supplementary Material of manuscript

Linking gas and particle ejection dynamics to boundary conditions in scaled shock-tube experiments

Valeria Cigala^1^, Ulrich Kueppers^1^, Juan Jose Peña Fernández^1^, Donald B. Dingwell^1^

1 Ludwig-Maximilians-Universität (LMU) Munich, Theresienstr 41, 80333 Munich, DE

Corresponding author: Valeria Cigala ([valeria.cigala@min.uni-muenchen.de](mailto:valeria.cigala@min.uni-muenchen.de))

Captions of supplementary figures uploaded separately and summary table.

**Fig. S1** Frame sequences from three different experiments to show the temporal evolution of the spreading angle, the numbers in the bottom sequence represent time in milliseconds (ms). To fit more frames in the image, each frame is cut at the center line of the vent exit. Time zero corresponds to the first appearance of condensed gas in the video, and it is not shown here. For all three sequences, common experimental conditions are cylinder vent geometry, 1-2 mm particle diameter and room temperature. The upper sequence shows setup 1; the middle sequence setup 2; the lower sequence setup 3. The same experimental conditions are shown in Fig. 7 and 8. It is possible to observe the delay in particle ejection for setup 1, between 5.8 to 6.8 ms, compared to setup 2 and 3, between 1.3 and 1.8 ms. Also, it is possible to observe how around 40 ms in setup 3 the few particles left exit the vent very sparsely.

**Fig. S2** Frame sequences from three different experiments to show the temporal evolution of the spreading angle, the numbers in the bottom sequence represent time in milliseconds (ms). To fit more frames in the image, each frame is cut at the center line of the vent exit. Time zero corresponds to the first appearance of condensed gas in the video, and it is not shown here. In the upper sequence the experimental conditions are cylinder vent geometry, 1-2 mm particle diameter, setup 1, room temperature. In the middle sequence the experimental conditions are funnel 15 vent geometry, 0.5-1 mm particle diameter, setup 2, high temperature. In the lower sequence the experimental conditions are nozzle vent geometry, 0.125-0.250 mm particle diameter, setup 3, high temperature. It is possible to observe the delay in particle ejection for setup 1, between 5.7 to 6.7 ms, compared to setup 2 and 3, between 1.2 and 1.7 ms. Also, it is possible to observe how around 40 ms the jet is not visible any more in the setup 3 sequence.

**Fig. S3** Gas and particle spreading angle evolution with time is shown here for the four different vent geometries and three different setups, SL 1–2 mm particles, 15 MPa, high temperature. The first panel for each setup includes a sketch of the setup where the part in colour represents where the particle sample is located. Time zero is defined as the first appearance of condensed gas in the video, the delay of particle exit is positively correlated with tube length (distance of sample surface from vent exit) before decompression. Error bars can be smaller than related symbol.

**Fig. S4** Gas and particle spreading angle evolution with time is shown here for the four different vent geometries and three different setups, SL 1–2 mm particles, 15 MPa, room temperature. The first panel for each setup includes a sketch of the setup where the part in colour represents where the particle sample is located. Time zero is defined as the first appearance of condensed gas in the video, the delay of particle exit is positively correlated with tube length (distance of sample surface from vent exit) before decompression. Error bars can be smaller than related symbol.

**Fig. S5** Gas and particle spreading angle evolution with time is shown here for the four different vent geometries and three different setups, SL 0.5-1 mm particles, 15 MPa, high temperature. The first panel for each setup includes a sketch of the setup where the part in colour represents where the particle sample is located. Time zero is defined as the first appearance of condensed gas in the video, the delay of particle exit is positively correlated with tube length (distance of sample surface from vent exit) before decompression. Error bars can be smaller than related symbol.

**Fig. S6** Gas and particle spreading angle evolution with time is shown here for the four different vent geometries and three different setups, SL 0.5-1 mm particles, 15 MPa, room temperature. The first panel for each setup includes a sketch of the setup where the part in colour represents where the particle sample is located. Time zero is defined as the first appearance of condensed gas in the video, the delay of particle exit is positively correlated with tube length (distance of sample surface from vent exit) before decompression. Error bars can be smaller than related symbol.

**Fig. S7** Gas and particle spreading angle evolution with time is shown here for the four different vent geometries and three different setups, SL 0.125-0.250 mm particles, 15 MPa, high temperature. The first panel for each setup includes a sketch of the setup where the part in colour represents where the particle sample is located. Time zero is defined as the first appearance of condensed gas in the video, the delay of particle exit is positively correlated with tube length (distance of sample surface from vent exit) before decompression. Error bars can be smaller than related symbol.

**Fig. S8** Gas and particle spreading angle evolution with time is shown here for the four different vent geometries and three different setups, SL 0.125-0.250 mm particles, 15 MPa, room temperature. The first panel for each setup includes a sketch of the setup where the part in colour represents where the particle sample is located. Time zero is defined as the first appearance of condensed gas in the video, the delay of particle exit is positively correlated with tube length (distance of sample surface from vent exit) before decompression. Error bars can be smaller than related symbol.

**Fig. S9** Gas spreading angle evolution of gas-only experiments performed at room temperature, with all vent geometries (see colour code legend on plot b.) for a. setup 2 and b. setup 3. In plot c. the spreading angle evolution of gas-only experiments performed with the cylinder geometry and setup 2 and 3 is compared to the trendlines of gas spreading angle for gas-particle experiments at room temperature, cylinder geometry, setup 1 (yellow line), setup 2 (blue line) and setup 3 (pink line), and coarse particles. Same trends shown on Figure 7 and 8 in the main text.

**Table S1**. Summary table of the experimental conditions performed and analysed for the present investigation on gas and particle spreading angle. The maximum gas spreading angle and the initial particle spreading angle values are provided for the left and right side of the gas and particle jets. *The maximum particle velocity represents the velocity of the fastest particle at ejection onset as measured by Cigala et al. (2017). **This experiment was randomly selected and three separate measurements performed on the same frames, therefore a standard deviation is provided for left and right side.

| **Exp Number** | **Sample origin** | **Grain size [mm]** | **Vent** | **T [°C]** | **Setup** | **v max particle [m/s] *** | **max θ° gas before particle ejection dx** | **max θ° gas before particle ejection sx** | **initial θ° particles dx** | **initial θ° particles sx** |
| --- | --- | --- | --- | --- | --- | --- | --- | --- | --- | --- |
| 1 | Schaumlava | 1-2 | Cylinder | 500 | 1 | 165 | 35.9 | 25.8 | 3.9 | 3.5 |
| 2 | Schaumlava | 1-2 | Cylinder | 500 | 1 | 195 | 32.8 | 31.3 | 5.3 | 1.9 |
| 3 | Schaumlava | 1-2 | Cylinder | 500 | 1 | 183 | 35.8 | 33.7 | 3.1 | 3.8 |
| 4 | Schaumlava | 0.5-1 | Cylinder | 500 | 1 | 180 | 27.4 | 36.9 | 3.0 | 3.7 |
| 5 | Schaumlava | 0.5-1 | Cylinder | 500 | 1 | 200 | 28.4 | 36.5 | 4.4 | 3.5 |
| 6 | Schaumlava | 0.5-1 | Cylinder | 500 | 1 | 182 | 36.5 | 36.5 | 3.0 | 4.7 |
| 7 | Schaumlava | 0.125-0.250 | Cylinder | 500 | 1 | NA | 37.4 | 27.2 | 3.3 | 4.1 |
| 8 | Schaumlava | 0.125-0.250 | Cylinder | 500 | 1 | NA | 28.8 | 35.5 | 5.3 | 5.3 |
| 9 | Schaumlava | 0.125-0.250 | Cylinder | 500 | 1 | NA | 34.7 | 34.2 | 4.7 | 3.5 |
| 10 | Schaumlava | 1-2 | Cylinder | 25 | 1 | 150 | 44.5 | 43.2 | 4.8 | 4.0 |
| 11 | Schaumlava | 1-2 | Cylinder | 25 | 1 | 135 | 41.9 | 45.9 | 5.1 | 4.5 |
| 12 | Schaumlava | 1-2 | Cylinder | 25 | 1 | 146 | 46.4 | 45.6 | 5.1 | 4.3 |
| 13 | Schaumlava | 0.5-1 | Cylinder | 25 | 1 | 165 | 44.1 | 45.0 | 3.6 | 3.9 |
| 14 | Schaumlava | 0.5-1 | Cylinder | 25 | 1 | 168 | 39.3 | 48.4 | 3.2 | 3.5 |
| 15 | Schaumlava | 0.5-1 | Cylinder | 25 | 1 | 156 | 44.3 | 39.6 | 3.4 | 4.7 |
| 16 | Schaumlava | 0.125-0.250 | Cylinder | 25 | 1 | NA | 36.3 | 35.5 | 6.5 | 5.6 |
| 17 | Schaumlava | 0.125-0.250 | Cylinder | 25 | 1 | NA | 41.1 | 36.0 | 6.2 | 4.6 |
| 18 | Schaumlava | 0.125-0.250 | Cylinder | 25 | 1 | NA | 36.3 | 38.7 | 5.4 | 5.4 |
| 19 | Schaumlava | 1-2 | Funnel 15 | 500 | 1 | 165 | 30.3 | 33.0 | 3.8 | 4.1 |
| 20 | Schaumlava | 1-2 | Funnel 15 | 500 | 1 | 174 | 31.2 | 25.1 | 3.9 | 4.2 |
| 21 | Schaumlava | 1-2 | Funnel 15 | 500 | 1 | 161 | 30.8 | 28.9 | 4.2 | 5.2 |
| 22 | Schaumlava | 0.5-1 | Funnel 15 | 500 | 1 | 192 | 34.1 | 33.7 | 3.5 | 4.4 |
| 23 | Schaumlava | 0.5-1 | Funnel 15 | 500 | 1 | 193 | 22.4 | 21.1 | 5.5 | 4.1 |
| 24 | Schaumlava | 0.5-1 | Funnel 15 | 500 | 1 | 203 | 29.7 | 33.7 | 4.1 | 3.5 |
| 25 | Schaumlava | 0.125-0.250 | Funnel 15 | 500 | 1 | NA | 31.2 | 20.8 | 3.1 | 3.5 |
| 26 | Schaumlava | 0.125-0.250 | Funnel 15 | 500 | 1 | NA | 25.8 | 33.7 | 3.6 | 4.5 |
| 27 | Schaumlava | 0.125-0.250 | Funnel 15 | 500 | 1 | NA | 25.9 | 26.1 | 3.8 | 4.2 |
| 28 | Schaumlava | 1-2 | Funnel 15 | 25 | 1 | 141 | 40.8 | 36.5 | 5.6 | 4.9 |
| 29 | Schaumlava | 1-2 | Funnel 15 | 25 | 1 | 137 | 37.6 | 29.4 | 5.5 | 5.1 |
| 30 | Schaumlava | 1-2 | Funnel 15 | 25 | 1 | 140 | 37.6 | 34.9 | 4.8 | 4.9 |
| 31 | Schaumlava | 0.5-1 | Funnel 15 | 25 | 1 | 192 | 35.9 | 35.0 | 4.7 | 4.0 |
| 32 | Schaumlava | 0.5-1 | Funnel 15 | 25 | 1 | 211 | 36.9 | 33.4 | 6.2 | 3.6 |
| 33 | Schaumlava | 0.5-1 | Funnel 15 | 25 | 1 | 180 | 40.2 | 39.3 | 5.2 | 3.1 |
| 34 | Schaumlava | 0.125-0.250 | Funnel 15 | 25 | 1 | NA | 35.5 | 34.8 | 4.7 | 4.8 |
| 35 | Schaumlava | 0.125-0.250 | Funnel 15 | 25 | 1 | NA | 34.7 | 33.7 | 4.6 | 5.6 |
| 36 | Schaumlava | 0.125-0.250 | Funnel 15 | 25 | 1 | NA | 32.7 | 38.7 | 6.0 | 5.6 |
| 37 | Schaumlava | 1-2 | Funnel 30° | 500 | 1 | 196 | 35.0 | 30.6 | 3.6 | 4.0 |
| 38 | Schaumlava | 1-2 | Funnel 30° | 500 | 1 | 192 | 30.8 | 30.2 | 3.8 | 4.7 |
| 39 | Schaumlava | 1-2 | Funnel 30° | 500 | 1 | 183 | 23.1 | 28.5 | 3.0 | 4.3 |
| 40 | Schaumlava | 0.5-1 | Funnel 30° | 500 | 1 | 166 | 22.1 | 33.0 | 2.4 | 3.1 |
| 41 | Schaumlava | 0.5-1 | Funnel 30° | 500 | 1 | 193 | 27.2 | 28.7 | 5.0 | 4.5 |
| 42 | Schaumlava | 0.5-1 | Funnel 30° | 500 | 1 | 188 | 27.4 | 35.5 | 2.5 | 4.1 |
| 43 | Schaumlava | 0.5-1 | Funnel 30° | 500 | 1 | 174 | 28.1 | 22.1 | 5.8 | 3.6 |
| 44 | Schaumlava | 0.125-0.250 | Funnel 30° | 500 | 1 | NA | 29.4 | 26.6 | 5.8 | 6.1 |
| 45 | Schaumlava | 0.125-0.250 | Funnel 30° | 500 | 1 | NA | 30.7 | 28.6 | 4.9 | 4.6 |
| 46 | Schaumlava | 0.125-0.250 | Funnel 30° | 500 | 1 | NA | 31.3 | 28.3 | 6.8 | 7.6 |
| 47 | Schaumlava | 1-2 | Funnel 30° | 25 | 1 | 155 | 34.2 | 39.4 | 5.6 | 5.6 |
| 48 | Schaumlava | 1-2 | Funnel 30° | 25 | 1 | 151 | 37.0 | 35.1 | 5.3 | 6.2 |
| 49 | Schaumlava | 1-2 | Funnel 30° | 25 | 1 | 160 | 34.7 | 37.3 | 5.3 | 5.6 |
| 50 | Schaumlava | 0.5-1 | Funnel 30° | 25 | 1 | 174 | 38.9 | 41.2 | 4.0 | 5.0 |
| 51 | Schaumlava | 0.5-1 | Funnel 30° | 25 | 1 | 171 | 41.1 | 33.7 | 5.2 | 3.2 |
| 52 | Schaumlava | 0.5-1 | Funnel 30° | 25 | 1 | 178 | 39.8 | 37.9 | 6.3 | 4.6 |
| 53 | Schaumlava | 0.125-0.250 | Funnel 30° | 25 | 1 | NA | 37.1 | 34.9 | 5.5 | 5.5 |
| 54 | Schaumlava | 0.125-0.250 | Funnel 30° | 25 | 1 | NA | 32.5 | 35.5 | 7.5 | 4.0 |
| 55 | Schaumlava | 0.125-0.250 | Funnel 30° | 25 | 1 | NA | 41.1 | 37.7 | 6.3 | 5.7 |
| 56 | Schaumlava | 1-2 | Convergent 5 | 500 | 1 | 137 | 43.0 | 36.9 | 2.2 | 4.2 |
| 57 | Schaumlava | 1-2 | Convergent 5 | 500 | 1 | 133 | 51.5 | 44.2 | 3.1 | 2.7 |
| 58 | Schaumlava | 1-2 | Convergent 5 | 500 | 1 | 129 | 47.2 | 43.7 | 3.4 | 4.2 |
| 59 | Schaumlava | 0.5-1 | Convergent 5 | 500 | 1 | 149 | 42.9 | 45.6 | 3.5 | 5.4 |
| 60 | Schaumlava | 0.5-1 | Convergent 5 | 500 | 1 | 142 | 42.7 | 46.2 | 5.3 | 5.7 |
| 61 | Schaumlava | 0.5-1 | Convergent 5 | 500 | 1 | 140 | 49.4 | 49.6 | 3.7 | 4.6 |
| 62 | Schaumlava | 0.125-0.250 | Convergent 5 | 500 | 1 | NA | 43.8 | 35.3 | 7.3 | 8.3 |
| 63 | Schaumlava | 0.125-0.250 | Convergent 5 | 500 | 1 | NA | 40.9 | 38.4 | 8.0 | 8.5 |
| 64 | Schaumlava | 0.125-0.250 | Convergent 5 | 500 | 1 | NA | 45.8 | 46.8 | 8.2 | 7.9 |
| 65 | Schaumlava | 1-2 | Convergent 5 | 25 | 1 | 116 | 43.7 | 41.6 | 6.5 | 5.1 |
| 66 | Schaumlava | 1-2 | Convergent 5 | 25 | 1 | 142 | 43.8 | 49.2 | 5.3 | 5.3 |
| 67 | Schaumlava | 1-2 | Convergent 5 | 25 | 1 | 131 | 45.0 | 51.1 | 6.4 | 4.2 |
| 68 | Schaumlava | 0.5-1 | Convergent 5 | 25 | 1 | 156 | 45.0 | 42.4 | 7.2 | 5.3 |
| 69 | Schaumlava | 0.5-1 | Convergent 5 | 25 | 1 | 145 | 40.9 | 40.4 | 5.4 | 5.6 |
| 70 | Schaumlava | 0.5-1 | Convergent 5 | 25 | 1 | 148 | 39.6 | 42.9 | 5.2 | 5.2 |
| 71 | Schaumlava | 0.125-0.250 | Convergent 5 | 25 | 1 | NA | 43.8 | 46.1 | 12.9 | 12.3 |
| 72 | Schaumlava | 0.125-0.250 | Convergent 5 | 25 | 1 | NA | 39.5 | 43.2 | 11.4 | 12.1 |
| 73 | Schaumlava | 0.125-0.250 | Convergent 5 | 25 | 1 | NA | 42.7 | 43.7 | 11.1 | 11.4 |
| 74 | Schaumlava | 1-2 | Cylinder | 500 | 2 | 190 | 40.9 | 40.5 | 10.6 | 10.0 |
| 75 | Schaumlava | 1-2 | Cylinder | 500 | 2 | 221 | 27.4 | 32.2 | 8.7 | 8.3 |
| 76 | Schaumlava | 1-2 | Cylinder | 500 | 2 | 251 | 32.2 | 34.5 | 6.7 | 6.7 |
| 77 | Schaumlava | 0.5-1 | Cylinder | 500 | 2 | 264 | 28.2 | 24.9 | 9.9 | 10.0 |
| 78 | Schaumlava | 0.5-1 | Cylinder | 500 | 2 | 254 | 30.7 | 25.9 | 9.7 | 8.1 |
| 79 | Schaumlava | 0.5-1 | Cylinder | 500 | 2 | 273 | 34.2 | 28.3 | 9.2 | 7.9 |
| 80 | Schaumlava | 0.125-0.250 | Cylinder | 500 | 2 | NA | 38.9 | 34.6 | 12.7 | 12.0 |
| 81 | Schaumlava | 0.125-0.250 | Cylinder | 500 | 2 | NA | 34.6 | 33.2 | 12.6 | 14.2 |
| 82 | Schaumlava | 0.125-0.250 | Cylinder | 500 | 2 | NA | 32.0 | 33.7 | 16.1 | 14.2 |
| 83 | Schaumlava | 1-2 | Cylinder | 25 | 2 | 215 | 37.8 | 38.8 | 11.1 | 11.3 |
| 84 | Schaumlava | 1-2 | Cylinder | 25 | 2 | 229 | 42.3 | 42.9 | 12.0 | 9.3 |
| 85 | Schaumlava | 1-2 | Cylinder | 25 | 2 | 219 | 44.5 | 41.6 | 10.1 | 9.9 |
| 86 | Schaumlava | 0.5-1 | Cylinder | 25 | 2 | 253 | 36.5 | 41.3 | 10.6 | 8.7 |
| 87 | Schaumlava | 0.5-1 | Cylinder | 25 | 2 | 251 | 37.5 | 32.9 | 9.5 | 9.1 |
| 88 | Schaumlava | 0.5-1 | Cylinder | 25 | 2 | 255 | 38.7 | 32.3 | 10.2 | 9.5 |
| 89 | Schaumlava | 0.125-0.250 | Cylinder | 25 | 2 | NA | 35.5 | 34.3 | 11.8 | 10.7 |
| 90 | Schaumlava | 0.125-0.250 | Cylinder | 25 | 2 | NA | 39.3 | 39.6 | 16.9 | 14.4 |
| 91 | Schaumlava | 0.125-0.250 | Cylinder | 25 | 2 | NA | 35.0 | 41.3 | 18.3 | 21.7 |
| 92 | Schaumlava | 1-2 | Funnel 15 | 500 | 2 | 251 | 25.2 | 25.9 | 6.4 | 6.0 |
| 93 | Schaumlava | 1-2 | Funnel 15 | 500 | 2 | 236 | 24.9 | 26.6 | 6.9 | 4.5 |
| 94 | Schaumlava | 1-2 | Funnel 15 | 500 | 2 | 283 | 29.0 | 28.4 | 6.5 | 6.6 |
| 95 | Schaumlava | 0.5-1 | Funnel 15 | 500 | 2 | 267 | 35.2 | 28.9 | 7.3 | 7.6 |
| 96 | Schaumlava | 0.5-1 | Funnel 15 | 500 | 2 | 296 | 20.2 | 30.7 | 9.7 | 7.9 |
| 97 | Schaumlava | 0.5-1 | Funnel 15 | 500 | 2 | 277 | 27.3 | 28.6 | 9.3 | 7.2 |
| 98 | Schaumlava | 0.125-0.250 | Funnel 15 | 500 | 2 | NA | 25.9 | 21.0 | 8.0 | 9.6 |
| 99 | Schaumlava | 0.125-0.250 | Funnel 15 | 500 | 2 | NA | 22.8 | 20.8 | 11.3 | 11.9 |
| 100 | Schaumlava | 0.125-0.250 | Funnel 15 | 500 | 2 | NA | 23.4 | 21.5 | 9.8 | 12.8 |
| 101 | Schaumlava | 1-2 | Funnel 15 | 25 | 2 | 219 | 26.6 | 34.7 | 7.6 | 6.7 |
| 102 | Schaumlava | 1-2 | Funnel 15 | 25 | 2 | 250 | 30.2 | 29.3 | 8.2 | 8.4 |
| 103 | Schaumlava | 1-2 | Funnel 15 | 25 | 2 | 259 | 36.0 | 30.6 | 5.6 | 7.1 |
| 104 | Schaumlava | 0.5-1 | Funnel 15 | 25 | 2 | 242 | 35.7 | 27.0 | 9.2 | 6.8 |
| 105 | Schaumlava | 0.5-1 | Funnel 15 | 25 | 2 | 249 | 28.5 | 25.3 | 7.4 | 6.1 |
| 106 | Schaumlava | 0.5-1 | Funnel 15 | 25 | 2 | 245 | 31.7 | 24.3 | 6.7 | 4.9 |
| 107 | Schaumlava | 0.125-0.250 | Funnel 15 | 25 | 2 | NA | 29.2 | 27.4 | 11.2 | 13.3 |
| 108 | Schaumlava | 0.125-0.250 | Funnel 15 | 25 | 2 | NA | 35.3 | 26.6 | 12.4 | 11.9 |
| 109 | Schaumlava | 0.125-0.250 | Funnel 15 | 25 | 2 | NA | 31.5 | 30.3 | 10.7 | 9.8 |
| 110 | Schaumlava | 1-2 | Funnel 30° | 500 | 2 | 235 | 26.1 | 27.0 | 8.2 | 8.8 |
| 111 | Schaumlava | 1-2 | Funnel 30° | 500 | 2 | 279 | 27.1 | 23.7 | 8.0 | 6.8 |
| 112 | Schaumlava | 1-2 | Funnel 30° | 500 | 2 | 247 | 27.6 | 20.1 | 8.6 | 7.6 |
| 113 | Schaumlava | 0.5-1 | Funnel 30° | 500 | 2 | 251 | 26.6 | 19.4 | 10.3 | 9.9 |
| 114 | Schaumlava | 0.5-1 | Funnel 30° | 500 | 2 | 252 | 32.7 | 27.3 | 8.9 | 10.4 |
| 115 | Schaumlava | 0.5-1 | Funnel 30° | 500 | 2 | 265 | 37.9 | 26.6 | 7.9 | 9.7 |
| 116 | Schaumlava | 0.125-0.250 | Funnel 30° | 500 | 2 | NA | 29.7 | 31.0 | 13.5 | 14.9 |
| 117 | Schaumlava | 0.125-0.250 | Funnel 30° | 500 | 2 | NA | 35.0 | 28.6 | 16.0 | 14.4 |
| 118 | Schaumlava | 0.125-0.250 | Funnel 30° | 500 | 2 | NA | 30.0 | 26.6 | 12.5 | 9.2 |
| 119 | Schaumlava | 1-2 | Funnel 30° | 25 | 2 | 226 | 28.7 | 28.2 | 10.4 | 11.1 |
| 120 | Schaumlava | 1-2 | Funnel 30° | 25 | 2 | 215 | 28.4 | 27.0 | 9.9 | 11.1 |
| 121 | Schaumlava | 1-2 | Funnel 30° | 25 | 2 | 234 | 29.2 | 34.0 | 10.3 | 10.7 |
| 122 | Schaumlava | 0.5-1 | Funnel 30° | 25 | 2 | 223 | 30.7 | 27.6 | 10.8 | 9.5 |
| 123 | Schaumlava | 0.5-1 | Funnel 30° | 25 | 2 | 229 | 28.8 | 28.5 | 11.1 | 9.0 |
| 124 | Schaumlava | 0.5-1 | Funnel 30° | 25 | 2 | 221 | 30.6 | 32.9 | 12.0 | 9.1 |
| 125 | Schaumlava | 0.125-0.250 | Funnel 30° | 25 | 2 | NA | 30.7 | 26.6 | 12.1 | 11.9 |
| 126 | Schaumlava | 0.125-0.250 | Funnel 30° | 25 | 2 | NA | 29.2 | 33.7 | 8.9 | 12.5 |
| 127 | Schaumlava | 0.125-0.250 | Funnel 30° | 25 | 2 | NA | 29.7 | 31.3 | 10.4 | 13.0 |
| 128 | Schaumlava | 1-2 | Convergent 5 | 500 | 2 | 243 | 31.2 | 24.9 | 13.7 | 14.8 |
| 129 | Schaumlava | 1-2 | Convergent 5 | 500 | 2 | 202 | 28.5 | 33.7 | 12.4 | 12.4 |
| 130 | Schaumlava | 1-2 | Convergent 5 | 500 | 2 | 232 | 29.0 | 31.6 | 11.0 | 12.7 |
| 131 | Schaumlava | 0.5-1 | Convergent 5 | 500 | 2 | 241 | 34.1 | 38.4 | 14.1 | 14.1 |
| 132 | Schaumlava | 0.5-1 | Convergent 5 | 500 | 2 | 211 | 39.3 | 33.2 | 13.5 | 12.5 |
| 133 | Schaumlava | 0.5-1 | Convergent 5 | 500 | 2 | 232 | 39.3 | 26.6 | 13.1 | 13.5 |
| 134 | Schaumlava | 0.125-0.250 | Convergent 5 | 500 | 2 | NA | 31.2 | 34.1 | 18.4 | 17.3 |
| 135 | Schaumlava | 0.125-0.250 | Convergent 5 | 500 | 2 | NA | 41.0 | 38.7 | 21.5 | 20.3 |
| 136 | Schaumlava | 0.125-0.250 | Convergent 5 | 500 | 2 | NA | 37.1 | 43.7 | 22.7 | 19.5 |
| 137 | Schaumlava | 1-2 | Convergent 5 | 25 | 2 | 204 | 45.7 | 45.8 | 13.5 | 12.7 |
| 138 | Schaumlava | 1-2 | Convergent 5 | 25 | 2 | 216 | 38.9 | 50.5 | 12.8 | 12.3 |
| 139 | Schaumlava | 1-2 | Convergent 5 | 25 | 2 | 219 | 41.9 | 45.0 | 16.3 | 13.7 |
| 140 | Schaumlava | 0.5-1 | Convergent 5 | 25 | 2 | 199 | 39.1 | 42.0 | 14.8 | 10.3 |
| 141 | Schaumlava | 0.5-1 | Convergent 5 | 25 | 2 | 226 | 38.5 | 42.0 | 12.9 | 16.6 |
| 142 | Schaumlava | 0.5-1 | Convergent 5 | 25 | 2 | 217 | 33.4 | 42.9 | 16.2 | 15.3 |
| 143 | Schaumlava | 0.125-0.250 | Convergent 5 | 25 | 2 | NA | 36.6 | 42.4 | 23.5 | 22.5 |
| 144 | Schaumlava | 0.125-0.250 | Convergent 5 | 25 | 2 | NA | 43.9 | 37.5 | 24.5 | 23.7 |
| 145 | Schaumlava | 0.125-0.250 | Convergent 5 | 25 | 2 | NA | 41.5 | 42.6 | 22.3 | 20.3 |
| 146 | Schaumlava | 1-2 | Cylinder | 25 | 3 | 185 | 42.7 | 35.7 | 4.2 | 2.8 |
| 147 | Schaumlava | 1-2 | Cylinder | 25 | 3 | 182 | 36.7 | 35.1 | 3.6 | 3.8 |
| 148 | Schaumlava | 1-2 | Cylinder | 25 | 3 | 168 | 40.2 | 36.7 | 4.2 | 4.5 |
| 149 | Schaumlava | 1-2 | Cylinder | 25 | 3 | NA | NA | NA | 4.7 | 3.1 |
| 150 | Schaumlava | 1-2 | Cylinder | 25 | 3 | NA | NA | NA | 3.5 | 4.4 |
| 151 | Schaumlava | 0.5-1 | Cylinder | 25 | 3 | 198 | 38.7 | 43.6 | 5.3 | 4.8 |
| 152 | Schaumlava | 0.5-1 | Cylinder | 25 | 3 | 206 | 37.6 | 29.9 | 4.9 | 3.6 |
| 153 | Schaumlava | 0.5-1 | Cylinder | 25 | 3 | 199 | 36.1 | 36.3 | 5.5 | 3.8 |
| 154 | Schaumlava | 0.5-1 | Cylinder | 25 | 3 | NA | NA | NA | 5.5 | 5.1 |
| 155 | Schaumlava | 0.125-0.250 | Cylinder | 25 | 3 | NA | 29.9 | 32.9 | 12.4 | 10.6 |
| 156 | Schaumlava | 0.125-0.251 | Cylinder | 25 | 3 | NA | 37.9 | 38.3 | 10.0 | 9.6 |
| 157 | Schaumlava | 0.125-0.252 | Cylinder | 25 | 3 | NA | 41.3 | 29.5 | 10.6 | 9.5 |
| 158 | Schaumlava | 1-2 | Cylinder | 500 | 3 | 204 | 29.2 | 22.8 | 6.0 | 4.9 |
| 159 | Schaumlava | 1-2 | Cylinder | 500 | 3 | 226 | 32.0 | 34.8 | 4.8 | 4.6 |
| 160 | Schaumlava | 1-2 | Cylinder | 500 | 3 | 220 | 31.0 | 30.6 | 4.5 | 5.0 |
| 161 | Schaumlava | 0.5-1 | Cylinder | 500 | 3 | 224 | 25.6 | 24.6 | 4.3 | 4.3 |
| 162 | Schaumlava | 0.5-1 | Cylinder | 500 | 3 | 241 | 33.9 | 21.3 | 6.8 | 5.6 |
| 163 | Schaumlava | 0.5-1 | Cylinder | 500 | 3 | 231 | 28.3 | 21.5 | 6.6 | 4.6 |
| 164 | Schaumlava | 0.125-0.250 | Cylinder | 500 | 3 | NA | 26.6 | 29.6 | 10.2 | 10.8 |
| 165 | Schaumlava | 0.125-0.250 | Cylinder | 500 | 3 | NA | 29.7 | 23.4 | 13.0 | 10.2 |
| 166 | Schaumlava | 0.125-0.250 | Cylinder | 500 | 3 | NA | 31.8 | 32.0 | 9.6 | 11.4 |
| 167 | Schaumlava | 1-2 | Funnel 15 | 25 | 3 | 198 | 31.8 | 31.4 | 7.1 | 5.1 |
| 168 | Schaumlava | 1-2 | Funnel 15 | 25 | 3 | 208 | 28.8 | 31.0 | 4.7 | 4.8 |
| 169 | Schaumlava | 1-2 | Funnel 15 | 25 | 3 | 199 | 28.3 | 32.9 | 4.3 | 3.8 |
| 170 | Schaumlava | 0.5-1 | Funnel 15 | 25 | 3 | 226 | 33.0 | 23.2 | 4.3 | 4.6 |
| 171 | Schaumlava | 0.5-1 | Funnel 15 | 25 | 3 | 234 | 28.0 | 31.4 | 4.0 | 4.3 |
| 172 | Schaumlava | 0.5-1 | Funnel 15 | 25 | 3 | 229 | 32.1 | 29.9 | 4.5 | 4.6 |
| 173 | Schaumlava | 0.125-0.250 | Funnel 15 | 25 | 3 | NA | 36.9 | 30.7 | 11.8 | 7.8 |
| 174 | Schaumlava | 0.125-0.250 | Funnel 15 | 25 | 3 | NA | 37.5 | 30.5 | 6.1 | 4.8 |
| 175 | Schaumlava | 0.125-0.250 | Funnel 15 | 25 | 3 | NA | 30.7 | 37.7 | 6.8 | 7.1 |
| 176 | Schaumlava | 1-2 | Funnel 15 | 500 | 3 | 277 | 26.6 | 24.1 | 4.8 | 5.0 |
| 177 | Schaumlava | 1-2 | Funnel 15 | 500 | 3 | 241 | 28.5 | 25.0 | 5.1 | 4.5 |
| 178 | Schaumlava | 1-2 | Funnel 15 | 500 | 3 | 219 | 21.3 | 23.9 | 6.0 | 4.4 |
| 179 | Schaumlava | 0.5-1 | Funnel 15 | 500 | 3 | 276 | 25.9 | 24.2 | 4.2 | 5.6 |
| 180 | Schaumlava | 0.5-1 | Funnel 15 | 500 | 3 | 247 | 29.6 | 23.2 | 6.9 | 5.9 |
| 181 | Schaumlava | 0.5-1 | Funnel 15 | 500 | 3 | 254 | 26.6 | 24.3 | 6.3 | 5.1 |
| 182 | Schaumlava | 0.125-0.250 | Funnel 15 | 500 | 3 | NA | 27.3 | 31.0 | 8.0 | 6.3 |
| 183 | Schaumlava | 0.125-0.250 | Funnel 15 | 500 | 3 | NA | 24.4 | 17.4 | 8.0 | 7.8 |
| 184 | Schaumlava | 0.125-0.250 | Funnel 15 | 500 | 3 | NA | 31.4 | 26.6 | 7.4 | 7.4 |
| 185 | Schaumlava | 1-2 | Funnel 30 | 25 | 3 | 174 | 27.1 | 30.0 | 5.3 | 6.5 |
| 186 | Schaumlava | 1-2 | Funnel 30 | 25 | 3 | 195 | 31.2 | 32.6 | 6.9 | 4.8 |
| 187 | Schaumlava | 1-2 | Funnel 30 | 25 | 3 | 184 | 27.4 | 22.8 | 7.7 | 6.1 |
| 188 | Schaumlava | 0.5-1 | Funnel 30 | 25 | 3 | 192 | 33.3 | 25.3 | 5.5 | 5.5 |
| 189 | Schaumlava | 0.5-1 | Funnel 30 | 25 | 3 | 181 | 31.0 | 32.2 | 4.4 | 5.1 |
| 190 | Schaumlava | 0.5-1 | Funnel 30 | 25 | 3 | 215 | 28.9 | 32.2 | 5.0 | 5.3 |
| 191 | Schaumlava | 0.125-0.250 | Funnel 30 | 25 | 3 | NA | 30.5 | 25.8 | 7.6 | 7.1 |
| 192 | Schaumlava | 0.125-0.250 | Funnel 30 | 25 | 3 | NA | 19.1 | 29.6 | 9.1 | 7.0 |
| 193 | Schaumlava | 0.125-0.250 | Funnel 30 | 25 | 3 | NA | 32.0 | 30.4 | 9.5 | 10.0 |
| 194 | Schaumlava | 1-2 | Funnel 30 | 500 | 3 | 246 | 20.9 | 25.9 | 5.1 | 6.5 |
| 195 | Schaumlava | 1-2 | Funnel 30 | 500 | 3 | 239 | 28.0 | 21.0 | 5.4 | 5.6 |
| 196 | Schaumlava | 1-2 | Funnel 30 | 500 | 3 | 243 | 27.9 | 25.6 | 4.6 | 5.9 |
| 197 | Schaumlava | 0.5-1 | Funnel 30 | 500 | 3 | 228 | 27.8 | 21.4 | 8.5 | 6.1 |
| 198 | Schaumlava | 0.5-1 | Funnel 30 | 500 | 3 | 233 | 28.2 | 23.5 | 7.9 | 6.7 |
| 199 | Schaumlava | 0.5-1 | Funnel 30 | 500 | 3 | 245 | 26.6 | 28.9 | 7.2 | 5.4 |
| 200 | Schaumlava | 0.125-0.250 | Funnel 30 | 500 | 3 | NA | 21.5 | 26.6 | 11.0 | 13.8 |
| 201 | Schaumlava | 0.125-0.250 | Funnel 30 | 500 | 3 | NA | 24.3 | 25.8 | 8.7 | 9.9 |
| 202 | Schaumlava | 0.125-0.250 | Funnel 30 | 500 | 3 | NA | 25.9 | 25.8 | 9.5 | 7.8 |
| 203 | Schaumlava | 1-2 | Convergent 5 | 25 | 3 | 177 | 47.7 | 41.6 | 7.0 | 6.9 |
| 204 | Schaumlava | 1-2 | Convergent 5 | 25 | 3 | 183 | 38.3 | 36.6 | 6.4 | 8.4 |
| 205 | Schaumlava | 1-2 | Convergent 5 | 25 | 3 | 185 | 34.8 | 41.8 | 7.1 | 6.4 |
| 206 | Schaumlava | 0.5-1 | Convergent 5 | 25 | 3 | 199 | 36.6 | 34.0 | 6.5 | 8.7 |
| 207 | Schaumlava | 0.5-1 | Convergent 5 | 25 | 3 | 226 | 40.2 | 36.3 | 5.6 | 6.8 |
| 208 | Schaumlava | 0.5-1 | Convergent 5 | 25 | 3 | 172 | 35.7 | 34.1 | 6.3 | 6.8 |
| 209 | Schaumlava | 0.125-0.250 | Convergent 5 | 25 | 3 | NA | 38.7 | 32.2 | 16.9 | 17.0 |
| 210 | Schaumlava | 0.125-0.250 | Convergent 5 | 25 | 3 | NA | 41.2 | 39.6 | 14.3 | 15.1 |
| 211 | Schaumlava | 0.125-0.250 | Convergent 5 | 25 | 3 | NA | 39.8 | 40.4 | 12.3 | 12.9 |
| 212 | Schaumlava | 1-2 | Convergent 5 | 500 | 3 | 212 | 39.3 | 27.4 | 6.0 | 5.8 |
| 213 | Schaumlava | 1-2 | Convergent 5 | 500 | 3 | 227 | 35.4 | 28.7 | 5.3 | 3.9 |
| 214 | Schaumlava | 1-2 | Convergent 5 | 500 | 3 | 190 | 37.3 | 30.1 | 6.1 | 4.4 |
| 215 | Schaumlava | 0.5-1 | Convergent 5 | 500 | 3 | 201 | 45.0 | 38.2 | 6.9 | 4.8 |
| 216 | Schaumlava | 0.5-1 | Convergent 5 | 500 | 3 | 194 | 40.4 | 30.3 | 8.0 | 6.2 |
| 217 | Schaumlava | 0.5-1 | Convergent 5 | 500 | 3 | 200 | 31.7 | 28.7 | 8.2 | 6.1 |
| 218 | Schaumlava | 0.125-0.250 | Convergent 5 | 500 | 3 | NA | 38.9 | 29.7 | 15.8 | 13.4 |
| 219 | Schaumlava | 0.125-0.250 | Convergent 5 | 500 | 3 | NA | 34.7 | 33.0 | 15.8 | 16.5 |
| 220 | Schaumlava | 0.125-0.250 | Convergent 5 | 500 | 3 | NA | 33.7 | 33.9 | 18.6 | 15.4 |
| 221 | Argon | NA | Convergent 5 | 25 | 2 | NA | 50.3(±1.5)** | 50(±4)** | 49.9 | 45 |
| 222 | Argon | NA | Cylinder | 25 | 2 | NA | 45.9 | 51.5 | NA | NA |
| 223 | Argon | NA | Funnel 15 | 25 | 2 | NA | 33.9 | 40.2 | NA | NA |
| 224 | Argon | NA | Funnel 30 | 25 | 2 | NA | 39.6 | 35.8 | NA | NA |
| 225 | Argon | NA | Convergent 5 | 25 | 3 | NA | 48 | 40.6 | NA | NA |
| 226 | Argon | NA | Cylinder | 25 | 3 | NA | 42.9 | 43.5 | NA | NA |
| 227 | Argon | NA | Funnel 15 | 25 | 3 | NA | 35.7 | 37.1 | NA | NA |
| 228 | Argon | NA | Funnel 30 | 25 | 3 | NA | 34.9 | 34.8 | NA | NA |
